# Synthesis and 2D-QSAR Study of Active Benzofuran-Based Vasodilators

**DOI:** 10.3390/molecules22111820

**Published:** 2017-10-26

**Authors:** Nagy M. Khalifa, Aladdin M. Srour, Somaia S. Abd El-Karim, Dalia O. Saleh, Mohamed A. Al-Omar

**Affiliations:** 1 Pharmaceutical Chemistry Department, Drug Exploration & Development Chair (DEDC), College of Pharmacy, King Saud University, Riyadh 11451, Saudi Arabia; nagykhalifa@hotmail.com (N.M.K.); Malomar1@KSU.EDU.SA (M.A.A.-O.); 2 Therapeutical Chemistry Department, National Research Centre, Dokki, Giza 12622, Egypt; somaia_elkarim@hotmail.com; 3 Pharmacology Department, National Research Centre, Dokki, Giza 12622, Egypt; doabdelfattah@yahoo.com

**Keywords:** benzofuran, vasodilation activity, 2D-QSAR, amiodarone hydrochloride

## Abstract

A new series of 2-alkyloxy-pyridine-3-carbonitrile-benzofuran hybrids (**4a**–**x**) was synthesized. All the new derivatives were examined via the standard technique for their vasodilation activity. Some of the investigated compounds exhibited a remarkable activity, with compounds **4w**, **4e**, **4r**, **4s**, **4f** and **4g** believed to be the most active hits in this study with IC_50_ values 0.223, 0.253, 0.254, 0.268, 0.267 and 0.275 mM, respectively, compared with amiodarone hydrochloride, the reference standard used (IC50 = 0.300 mM). CODESSA PRO was employed to obtain a statistically significant 2-Dimensional Quantitative Structure Activity Relationship (2D-QSAR) model describing the bioactivity of the newly synthesized analogs (*N* = 24, *n* = 4, *R*^2^ = 0.816, *R*^2^_cv_OO = 0.731, *R*^2^_cv_MO = 0.772, *F* = 21.103, *s*^2^ = 6.191 × 10^−8^).

## 1. Introduction

**Figure 1:**
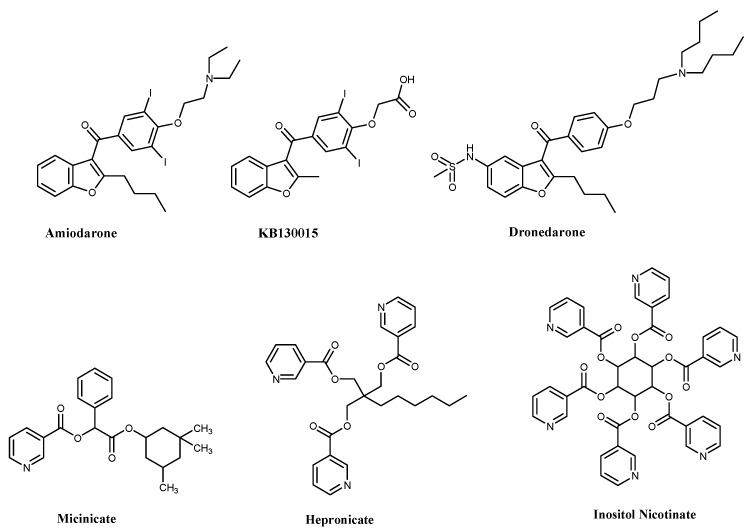
Structures of some active vasodilating agents.

Hypertension is the most common cardiovascular and cerebrovascular disorder representing the major risk factor for endothelial dysfunctions [1,2]. Worldwide, one of every three adults is reported to have high blood pressure, which is responsible for half of the mortalities related to stroke and heart disease [2,3]. Under hypertensive conditions, many functional organs can suffer irreparable lesions [4,5]. Essential hypertension is a common trait caused by many factors and it increases the risk of cardiovascular (heart attacks), cerebrovascular (stroke), peripheral artery, rheumatic heart, congenital heart, heart failure and renal diseases [6,7,8]. A benzofuran-containing compound, amiodarone, is one of the most therapeutically important antiarrhythmic drugs for various types of cardiac dysrhythmias [9,10] (Figure 1). Though the responsible pharmacological mechanisms of amiodarone’s antiarrhythmic effects are not settled [11], it has an extreme effect on various ionic currents [12], as well as sodium, calcium and potassium fluxes. These actions are interrelated in a complex way, but are of prime importance for its activity. Amiodarone also possesses coronary and peripheral vasodilator properties [11]—this appears to be mainly due to a release of nitric oxide (NO). Moreover, it expands the precompressed in vivo human hand veins through the activation of NO synthase and blockade of α-adrenergic mechanisms as a venodilator [13,14], and amiodarone’s analog KB130015 (Figure 1) activates the BK_Ca_ channels, which relaxes vascular smooth muscle cells. KB130015 is a novel BK_Ca_ activator—its efficacy is based on the subunit composition of the channel complex [15]. Dronedarone as well as KB130015 (Figure 1) is a noniodinated congener of amiodarone that has been developed and approved by FDA (Food and Drug Administration) to avoid the limiting iodine-associated adverse effects of the commercially used amiodarone. Additionally, dronedarone displays antiadrenergic properties, atrial flutter and atrial fibrillation [16]. Consequently, the stimulation of coronary dilation by dronedarone involving a dual mechanism, putative Ca^2+^ channels inhibition and stimulation of NO synthase pathway [16,17]. Benzofurans are naturally existing scaffolds [18], associated with a broad range of chemotherapeutic properties [19,20,21,22,23,24,25]. Nicotinate esters are very interesting vasodilatory active heterocycles [26,27], also, many nicotinate analogs such as, micinicate, hepronicate and inositol nicotinate are of significant vasodilating activity [28], (Figure 1).

In the present study, we designed and synthesized some novel hybrids of pyridine-3-carbonitriles and benzofuran-pyrazole functions, attributed to the fact that, pyridine-3-carbonitriles are interesting agents in developing new active hits due to the recognition of bioisosterism with the nicotinate analogs where the acid/ester function is just replaced by a cyano group [28]. Furthermore, it is known that the benzofuran-pyrazole hybrid is of considerable vasorelaxant interest [29], this may due to the belief that the aliphatic secondary amine side chain of amiodarone might be responsible for its vasodilation activity [30,31]. Thus, the insertion of a pyrazole ring system in this scaffold may widen new pharmacological active hits with higher potency and fewer side effects. In addition, studying the two-dimensional quantitative structure activity relationships (2D-QSAR) for the newly synthesized analogs explored the controlling factors governing the observed pharmacological properties as well as validated of the observed activity of the new chemical entities.

## 2. Results and Discussion

### 2.1. Chemistry

**Scheme 1:**
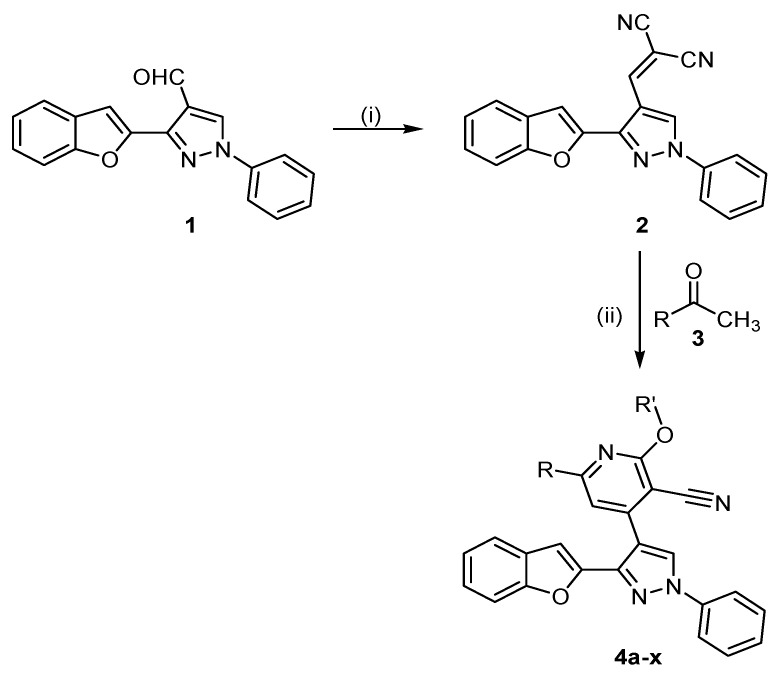
Synthetic route of 2-alkyloxy-pyridine-3-carbonitrile derivatives **4a**–**x**.

The assumed synthetic approach to obtain target derivatives is illustrated in (Scheme 1). Treating 2-acetylbenzofuran, the key starting compound in this study, with phenyl hydrazine afforded the corresponding semihydrazone derivative which undergo formylation via Vilsmeier–Haack reaction to give 3-(benzofuran-2-yl)-1-phenyl-1*H*-pyrazole-4-carbaldehyde (**1**) [32]. The formed carbaldehyde **1** was treated with malononitrile in refluxing ethanol to give 2-((3-(benzofuran-2-yl)-1-phenyl-1*H*-pyrazol-4-yl)-methylene)malononitrile (**2**). Adopting the reported procedure [32], the required 2-alkyloxy-pyridine-3-carbonitrile derivatives **4a**–**x** were synthesized via the condensation reaction of aromatic ketones **3a**–**l** with ylidenemalononitrile **2** and sodium alkoxide of the corresponding alcohol (Scheme 1). As a representative example, the IR spectrum of compound **4a** shows a strong stretching vibration band at ν = 2213 cm^−1^ for nitrile group. ^1^H-NMR spectrum of **4a** exhibits a characteristic signal at δ = 4.23 ppm refering to the methoxide group, while the pyridinyl H-5 appeared as singlet peak at δ = 8.42 ppm. ^13^C-NMR spectrum of **4a** reveals the presence of a methoxide carbon at δ = 54.7 ppm, pyridinyl C-3, C-5 and nitrile carbon signals appeared at δ = 93.5, 105.8, and 114.6 ppm, respectively. Mass spectrum (EI) of **4a** reveals the molecular ion peak 482.27 with relative intensity value 30.7%. The established structures of all new chemical entities **4a**–**x** were certified by their microanalyses and spectral data (IR, ^1^H-NMR, ^13^C-NMR and EI-MS).

### 2.2. Biological Evaluation

#### 2.2.1. Vasodilation Properties

Vasodilation properties of the synthesized 2-alkyloxy-pyridine-3-carbonitrile derivatives **4a**–**x** were inspected applying the separated thoracic aortic rings of mice pre-contracted by norepinephrine hydrochloride according to the standard method [33] using amiodarone hydrochloride as a standard reference. The observed data (Table 1), (Figures S1 and S2 of Supplementary Materials) reveal that most the new chemical entities reveal remarkable vasodilation properties. Meanwhile, compounds **4w**, **4e**, **4r**, **4s**, **4f** and **4g** exhibit significant activity (IC_50_, is the required concentration for 50% lessening of maximal norepinephrine. HCl induced contracture = 0.223, 0.253, 0.254, 0.268, 0.267 and 0.275 mM, respectively), that seems more potent than the used reference standard in the present study (IC_50_ = 0.300 mM). The 2D-QSAR study was initiated to recognize the observed bioactivities and concluding the most important factors that manage the pharmacological properties. Currently, throughout the observed vasodilator activities of the new chemical entities, few Structure activity relationship (SAR) rules could be achieved, the presence of a methoxy group at the 2-position of 3-pyridinecarbonitriles enhances the vasodilation activity more than the ethoxy group, as shown in all of the tested analogs. Compounds **4k** and **4q** (IC_50_ = 0.428, 0.321 mM, respectively) are exceptions. Benzimidazole ring systems attached to pyridine-C_5_ seems appropriate for designing vasodilation active hits (IC_50_ = 0.223, 0.299 mM) compared with the corresponding substituted phenyl ring systems or other heterocycles. Thus, the combination of benzimidazole, pyridine and benzofuran has the potential to be developed into potent vasorelaxant active targets.

**Figure 2:**
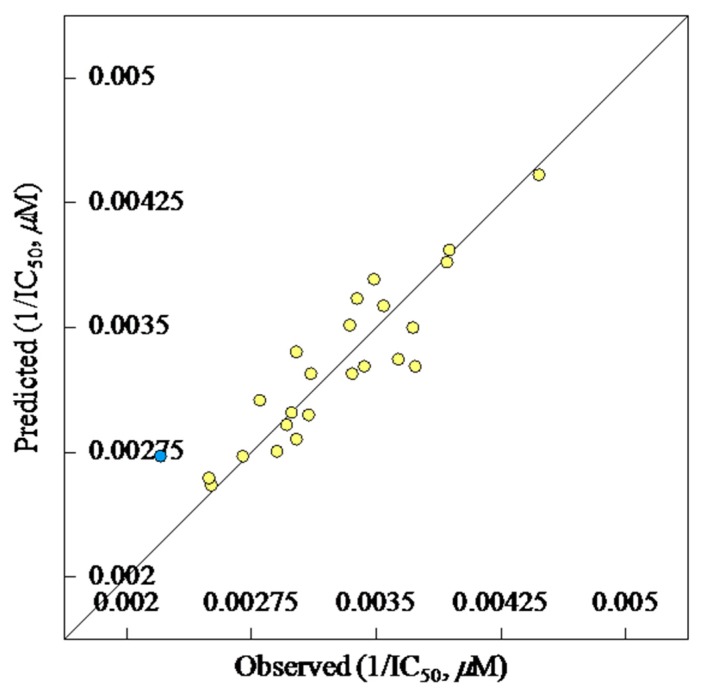
BMLR-QSAR model plot of correlations, the yellow dots are representing the observed vs. predicted vasodilatory active agents for all compounds, while the blue dot is represent an outlier compound (compound **4j**).

To validate and understand the observed pharmacological activities and to detect the factors that control the activities, the 2D-QSAR study was initiated via the CODESSA PRO package. Molecular descriptors of the 2D-QSAR correlating the chemical structure(s) and property values expressed as 1/IC_50_ µM are presented in Table 2, arranged on their level of significance (*t*-criterion). The descriptors were acquired using the BMLR (Best Multiple Linear Regression) method. The first descriptor controlling the BMLR-QSAR model based on its *t*-criterion value (*t* = 7.789) is maximum e–e repulsion for bond C–O which is a semiempirical descriptor. Electron–electron repulsion between two given atoms is determined by Equation (1) [34].

(1) $$E_{ee}(AB)=\sum_{\mu ,\nu \in A}\sum_{\lambda ,\sigma \in B}P_{\mu \nu }P_{\lambda \sigma }\langle \mu \nu |\lambda \sigma \rangle$$

where, *A* stands for a given atomic species, *B* is another atomic species *P_μ_*_ν_, *P_λσ_* is density matrix elements over atomic basis {*μ*ν*λσ*}, 〈μν|λσ〉 is the electron repulsion integrals on atomic basis {*μ*ν*λσ*}. The second important descriptor controlling the BMLR-QSAR model (*t* = −3.637) is surface-weighted charged partial-negative charged surface area (*WNSA1*) weighted PNSA (*PNSA1* × *TMSA*/1000) (MOPAC PC) (charge-related descriptor). Surface-weighted charged partial negative-charged surface area (*WNSA1*) is calculated by Equation (2) [34].

(2) $$WNSA1=\frac{PNSA1\cdot TMSA}{1000}$$

where, *PNSA1* stands for partial negatively charged molecular surface area, *TMSA* for total molecular surface area. The third descriptor controlling BMLR-QSAR model (*t* = −5.670) is the fractional hydrogen bonding acceptor ability of the molecule *FHACA1,* which is also a charge-related descriptor determined by Equation (3) [34].

(3) $$FHACA1=\frac{HACA1}{TMSA}$$

where, *HACA1* is hydrogen bonding acceptor ability, *TMSA* is the total molecular surface area. The fourth descriptor controlling BMLR-QSAR model (*t* = −6.241) is a semiempirical descriptor, maximum e–n attraction for bond C–N, is determined by Equation (4) [34].

(4) $$E_{ne}\left(AB\right)=\sum_{B}\sum_{\mu ,\nu \in A}P_{\mu \nu }\langle \mu |\frac{Z_{B}}{R_{iB}}|\nu \rangle$$

where, *A* stands for a given atomic species, *B* is another atomic species *P_μ_*_ν_ is density matrix elements over atomic basis {*μ*ν}, *Z_B_* for charge of atomic nucleus *B*, *R_iB_* for distance between the electron and atomic nucleus *B*, $\langle \mu |\frac{Z_{B}}{R_{iB}}|\nu \rangle$ for electron–nuclear attraction integrals on atomic basis {*μ*ν}. The correlation between the observed and predicted vasodilation activities is represented in Figure 2. The descriptor values for each respective compound are exhibited in Table S1 of Supplementary Material.

The reliability and statistical relevance of the attained BMLR-QSAR model is examined by internal validation technique, which is an appropriate technique due to the limited data points of the present study [35,36,37]. Internal validation is applied by the CODESSA PRO employing both Leave One Out (LOO), which involves developing a number of models with one example omitted at a time, and Leave Many Out (LMO) that develops a number of models with many data points omitted at a time (up to 20% of the total data points). The observed correlations attributed to the internal validation techniques are *R*^2^_cv_OO = 0.731, *R*^2^_cv_MO = 0.772. Both of them are significantly correlated with the R^2^value of the attained QSAR model (*R*^2^ = 0.816). Standard deviation of the regressions (*s*^2^ = 6.191 × 10^−8^) and Fisher test value (*F* = 21.103) are also statistical parameters supporting the QSAR model. The predicted/estimated IC_50_ values of the new chemical entities according to the achieved BMLR-QSAR model are displayed in Table 3. The obtained results revealed that the most potent analogue among all the tested hybrids, compound **4w**, shows error value (difference between estimated and observed IC_50_ values) = −0.4. Additionally, the high potent analogues synthesized **4a**–**i**, relative to the standard reference, amiodarone hydrochloride (IC_50_ = 300 µM), also exhibited estimated bioproperties matched with their observed potencies (IC_50_ = 253–397 µM, 253–392 µM, corresponding to the observed and predicted values respectively, error = 0–−40). Compound **4j** is the only exception with high error value = 83 with (IC_50_ = 452, 369 µM, for observed and predicted bio-data, respectively). This compound is exhibited as an outlier (Figure 2) and shows the lowest vasodilation properties among all the synthesized hybrids. From all the above, it can be concluded that the achieved BMLR-QSAR model is statistically significant and also supported by the matched correlations due to the observed and predicted bio-observations. Success of this study can be attributed to the homogeneity of chemical structural entities. Additionally, the achieved model can be adopted for optimizing hits of high potency relative to the standard reference used based on the hybrid design mentioned in the present study.

#### 2.2.2. Toxicological Bioassay

The most potent hits in this study, compounds (**4a**, **c**, **e**, **f**, **g**, **l**, **m**, **r**, **s**,**t**, **w** and **4x**), were tested at 1000 mg kg^−1^ (mouse body weight), with no toxic symptoms or mortality rates being observed after 24 h post-administrations elucidating the safe behavior of the used doses. Thus, the present study recommended that the benzofuran-containing compounds may have the potential to be developed into potent vasodilatory active agents.

## 3. Experimental Section

### 3.1. General Information

Melting points were recorded on a Stuart SMP30 melting point apparatus. IR spectra (KBr) were recorded on a JASCO 6100 spectrophotometer, JASCO, Easton, USA. NMR spectra were recorded on a JEOL AS 500 (DMSO-*d*_6_, ^1^H: 500 MHz, ^13^C: 125 MHz) spectrometer, JEOL USA, Inc. (Pleasanton, CA, USA). Chemical shifts (δ_H_) are reported relative to Tetramethylsilane (TMS) as the internal standard. All coupling constant (*J*) values are given in hertz. Chemical shifts (δ_c_) are reported relative to CDCl_3_ as internal standards. Mass spectra were recorded on a Shimadzu GCMS-QP 1000 EX (EI, 70 eV) spectrometer, Shimadzu corporation, Kyoto, Japan. Elemental microanalyses were performed by using a Vario Elemental analyzer, Elementar Analysensysteme GmbH, Langenselbold, Germany. 2-Acetylbenzofuran [38], 3-(benzofuran-2-yl)-1-phenyl-1*H*-pyrazole-4-carbaldehyde (**1**) [32] and 2-((3-(benzofuran-2-yl)-1-phenyl-1*H*-pyrazol-4-yl)methylene)malononitrile (**1**) [32] were prepared according to the previously reported procedures.

#### 3.1.1. Synthesis of 2-((3-(Benzofuran-2-yl)-1-phenyl-1*H*-pyrazol-4-yl)methylene)malononitrile (**2**)

The formed carbaldehyde **1** (2.88 g, 10 mmol) was stirred in ethanol at room temperature (25–30 °C) for 6 h with malononitrile (0.66 g, 10 mmol) in the presence of few drops of piperidine, the formed precipitate was filtered, dried and recrystallized from *n*-butanol, to afford 2.54 g of compound **2** (75% yield).

#### 3.1.2. Synthesis of 2-Alkoxy-4-(3-(benzofuran-2-yl)-1-phenyl-1*H*-pyrazol-4-yl)-6-phenylpyridine-3-carbonitriles (**4a**–**x**)

##### General Procedure

A mixture of equimolar amounts of **2** (3.36 g, 10 mmol) and methyl aryl ketones **3a**–**l** (10 mmol), in the appropriate alcohol (20 mL) containing sodium (0.46 g, 20 mmol) was stirred at room temperature (25–30 °C) for the proper time controlled by Thin-layer chromatography (TLC). The solid separated was collected, washed with water and crystallized from *n*-butanol to afford the title compounds **4a**–**x**.

*4-(3-(Benzofuran-2-yl)-1-phenyl-1H-pyrazol-4-yl)-2-methoxy-6-phenylpyridine-3-carbonitrile* (**4a**): yield 1.86 g (39.7%), m.p. 198–200 °C. IR: ν_max_/cm^−1^ 2213 (C≡N), 1588, 1543 (C=N, C=C). ^1^H-NMR (CDCl_3_): δ 4.23 (s, 3H), 7.08 (s, 1H), 7.25–7.29 (m, 4H), 7.39–8.04 (m, 6H), 7.09 (d, *J* = 7.7 Hz, 2H), 8.04 (d, *J* = 7.7 Hz, 2H), 8.38 (s, 1H), 8.42 (s, 1H). ^13^C-NMR (CDCl_3_): δ 54.7 93.5, 105.7, 105.8, 111.6, 111.7, 114.6, 119.8, 120.0, 121.4, 123.3, 125.1, 126.7, 127.4, 127.8, 127.9, 128.4, 128.6, 128.7, 129.0, 129.7, 130.6, 139.3, 154.9, 155.1, 157.8, 165.0. MS: *m*/*z* (%) 468.31 (M, 0.47). Anal. for C_30_H_20_N_4_O_2_ (468.51); Calcd. C, 76.91; H, 4.30; N, 11.96. Found: C, 76.84; H, 4.26; N, 11.87.

*4-(3-(Benzofuran-2-yl)-1-phenyl-1H-pyrazol-4-yl)-2-ethoxy-6-phenylpyridine-3-carbonitrile* (**4b**): yield 1.84 g (39%), m.p. 186–188 °C. IR: ν_max_/cm^−1^: 2215 (C≡N), 1590, 1541 (C=N, C=C). ^1^H-NMR (CDCl_3_): δ 1.55 (t, *J* = 7.6 Hz, 3H), 4.69 (q, *J* = 7.6 Hz, 2H), 7.05 (s, 1H), 7.20–7.53 (m, 10 H), 7.81–8.10 (m, 4 H), 8.40 (s, 1H), 8.50 (s, 1H). ^13^C-NMR (CDCl_3_): δ 14.8, 63.5, 93.6, 105.8, 111.6, 114.3, 116.1, 118.1, 119.0, 120.0, 121.4, 123.3, 125.1, 127.3, 127.8, 128.6, 128.7, 128.9, 129.7, 132.5, 135.4, 138.5, 140.0, 143.0, 147.3, 148.9, 155.1, 157.7, 164.8. MS: *m*/*z* (%) 482.25 (M, 20.34). Anal. for C_31_H_22_N_4_O_2_ (482.53); Calcd. C, 77.16; H, 4.60; N, 11.61. Found: C, 77.05; H, 4.57; N, 11.72.

*4-(3-(Benzofuran-2-yl)-1-phenyl-1H-pyrazol-4-yl)-6-(4-chlorophenyl)-2-methoxypyridine-3-carbonitrile* (**4c**): yield 2.04 g (40.5%), m.p. 259–261 °C. IR: ν_max_/cm^−1^ 2214 (C≡N), 1588, 1542 (C=N, C=C). ^1^H-NMR (DMSO-*d*_6_): δ 4.14 (s, 3H), 7.08 (s, 1H), 7.20–7.58 (m, 9H), 7.92 (s, 1H), 8.0 (d, *J* = 8 Hz, 2H), 8.12 (d, *J* = 8 Hz, 2H), 9.1 (s, 1H). MS: *m*/*z* (%) 502.11 (M, 0.47). Anal. for C_30_H_19_ClN_4_O_2_ (502.95); Calcd. C, 71.64; H, 3.81; N, 11.14. Found: C, 71.56; H, 3.78; N, 11.18.

*4-(3-(Benzofuran-2-yl)-1-phenyl-1H-pyrazol-4-yl)-6-(4-chlorophenyl)-2-ethoxypyridine-3-carbonitrile* (**4d**): yield 2.27 g (40.4%), m.p. 199–201 °C. IR: ν_max_/cm^−1^ 2219 (C≡N), 1588, 1541 (C=N, C=C). ^1^H-NMR (CDCl_3_): δ 1.54 (t, *J* = 6.7 Hz, 3H), 4.67 (q, *J* = 6.7 Hz, 2H), 7.08 (s, 1H), 7.35–7.53 (m, 10H), 7.81–7.84 (m, 4H), 8.43 (s, 1H). ^13^C-NMR (CDCl_3_): δ 14.6, 63.5, 93.8, 105.9, 111.5, 114.1, 116.0, 117.5, 119.9, 121.5, 123.4, 125.2, 127.9, 128.4, 128.6, 129.0, 129.2, 129.8, 135.8, 136.7, 139.2, 148.9, 149.0, 154.9, 156.4, 164.7. MS: *m*/*z* (%) 516.30, 518.30 (M, M+2, 1.95, 0.68). Anal. for C_31_H_21_ClN_4_O_2_ (516.98); Calcd. C, 72.02; H, 4.09; N, 10.84. Found: C, 72.14; H, 4.14; N, 10.78.

*4-(3-(Benzofuran-2-yl)-1-phenyl-1H-pyrazol-4-yl)-6-(4-bromophenyl)-2-methoxypyridine-3-carbonitrile* (**4e**): yield 2.14 g (39%), m.p. 273–275 °C. IR: ν_max_/cm^−1^ 2217 (C≡N), 1585, 1542 (C=N, C=C). ^1^H-NMR (DMSO-*d*_6_): δ 4.14 (s, 3H), 7.09 (s, 1H), 7.20–7.73 (m, 10H), 7.96–7.16 (m, 4H), 9.10 (s, 1H). MS: *m*/*z* (%) 546.16 (M, 3.22). Anal. for C_30_H_19_BrN_4_O_2_ (547.40): Calcd. C, 65.82; H, 3.50; N, 10.24. Found: C, 65.94; H, 3.38; N, 10.17.

*4-(3-(Benzofuran-2-yl)-1-phenyl-1H-pyrazol-4-yl)-6-(4-bromophenyl)-2-ethoxypyridine-3-carbonitrile* (**4f**): yield 1.94 g (34.6%), m.p. 207–209 °C. IR: ν_max_/cm^−1^ 2219 (C≡N), 1588, 1540 (C=N, C=C). ^1^H-NMR (CDCl_3_): δ 1.54 (t, *J* = 6.7 Hz, 3H), 4.67 (q, *J* = 6.7 Hz, 2H), 7.09 (s, 1H), 7.20–7.52 (m, 10H), 7.77–7.81 (m, 4H), 8.44 (s, 1H). ^13^C-NMR (CDCl_3_): δ 14.6, 63.6, 93.9, 105.9, 111.5, 114.1, 115.7, 117.5, 120.0, 121.5, 123.4, 125.2, 127.9, 128.4, 128.8, 129.0, 129.8, 132.2, 136.3, 139.3, 142.4, 147.4, 148.9, 154.9, 156.5, 164.8. MS: *m*/*z* (%) 560.40 (M, 0.24). Anal. for C_31_H_21_BrN_4_O_2_ (561.43); Calcd. C, 66.32; H, 3.77; N, 9.98. Found: C, 66.54; H, 3.84; N, 9.82.

*4-(3-(benzofuran-2-yl)-1-phenyl-1H-pyrazol-4-yl)-6-(4-fluorophenyl)-2-methoxypyridine-3-carbonitrile* (**4g**): yield 1.96 g (40.3%), m.p. 237–239 °C. IR: ν_max_/cm^−1^ 2215 (C≡N), 1590, 1543 (C=N, C=C). ^1^H-NMR (CDCl_3_): δ 4.22 (s, 3H), 7.07 (s, 1H), 7.25–7.53 (m, 10H), 7.81–7.93 (m, 4H), 8.44 (s, 1H).MS: *m*/*z* (%) 486.31 (M, 1.90). Anal. for C_30_H_19_FN_4_O_2_ (486.5); Calcd. C, 74.06; H, 3.94; N, 11.52. Found: C, 73.95; H, 3.88; N, 11.48.

*4-(3-(Benzofuran-2-yl)-1-phenyl-1H-pyrazol-4-yl)-2-ethoxy-6-(4-fluorophenyl)pyridine-3-carbonitrile* (**4h**): yield 2.12 g (42.4%), m.p. 204–206 °C. IR: ν_max_/cm^−1^ 2218 (C≡N), 1592, 1543 (C=N, C=C). ^1^H-NMR (CDCl_3_): δ 1.53 (t, *J* = 7 Hz, 3H), 4.67 (q, *J* = 7 Hz, 2H), 7.08 (s, 1H), 7.39–7.54 (m, 10 H), 7.81–7.83 (m, 4H), 8.44 (s, 1H). ^13^C-NMR (CDCl_3_): δ 14.6, 63.6, 93.5, 105.9, 111.5, 115.9, 116.1, 120.0, 121.4, 123.4, 125.1, 127.9, 129.0, 129.3, 129.4, 129.8, 139.3, 142.4, 147.4, 148.9, 154.9, 156.6, 163.3, 164.8, 165.3. MS: *m*/*z* (%) 500.36 (M, 2.71). Anal. for C_31_H_21_FN_4_O_2_ (500.52); Calcd. C, 74.39; H, 4.23; N, 11.19. Found: C, 74.45; H, 4.18; N, 11.14.

*4-(3-(Benzofuran-2-yl)-1-phenyl-1H-pyrazol-4-yl)-2-methoxy-6-p-tolylpyridine-3-carbonitrile* (**4i**): yield 2.3 g (47.7%), m.p. 258–260 °C. IR: ν_max_/cm^−1^ 2218 (C≡N), 1588, 1541 (C=N, C=C). ^1^H-NMR (CDCl_3_): δ 2.39 (s, 3H), 4.22 (s, 3H), 7.06 (s, 1H), 7.22–7.52 (m, 10H), 7.58–7.86 (m, 4H), 8.41 (s, 1H). ^13^C-NMR (CDCl_3_): δ 21.5, 54.7, 93.1, 105.8, 111.6, 114.2, 115.9, 117.6, 120.1, 121.4, 123.3, 125.1, 127.3, 127.9, 128.4, 129.0, 129.7, 134.5, 139.3, 141.0, 147.2, 148.9, 154.5, 157.9, 165.0. MS: *m*/*z* (%) 482.27 (M, 30.7). Anal. for C_31_H_22_N_4_O_2_ (482.53): Calcd. C, 77.16; H, 4.60; N, 11.61. Found: C, 77.29; H, 4.42; N, 11.51.

*4-(3-(Benzofuran-2-yl)-1-phenyl-1H-pyrazol-4-yl)-2-ethoxy-6-p-tolylpyridine-3-carbonitrile* (**4j**): yield 1.92 g (38.7%), m.p. 202–204 °C. IR: ν_max_/cm^−1^ 2215 (C≡N), 1598, 1543 (C=N, C=C). ^1^H-NMR (CDCl_3_): δ 1.54 (t, *J* = 6 Hz, 3H), 2.39 (s, 3H), 4.69 (q, *J* = 6 Hz, 2H), 7.05 (s, 1H), 7.20–7.56 (m, 10H), 7.81–7.85 (m, 4H), 8.41 (s, 1H). ^13^C-NMR (CDCl_3_): δ 14.7, 21.5, 63.4, 93.2, 105.8, 111.6, 114.0, 116.0, 117.7, 120.0, 121.4, 123.3, 125.1, 127.3, 127.8, 128.4, 129.0, 129.7, 134.6, 139.3, 141.0, 143.0, 147.2, 149.0, 154.9, 157.8, 164.7. MS: *m*/*z* (%) 496.22 (M, 5.52). Anal. for C_32_H_24_N_4_O_2_ (496.56): Calcd. C, 77.40; H, 4.87; N, 11.28. Found: C, 77.52; H, 4.76; N, 11.34.

*4-(3-(Benzofuran-2-yl)-1-phenyl-1H-pyrazol-4-yl)-2-methoxy-6-(4-methoxyphenyl)pyridine-3-carbonitrile* (**4k**): yield 1.86 g (37.3%), m.p. 256–258 °C. IR: ν_max_/cm^−1^ 2213 (C≡N), 1588, 1543 (C=N, C=C). ^1^H-NMR (DMSO-*d*_6_): δ 3.80 (s, 3H), 4.13 (s, 3H), 7.04 (s, 1H), 7.20–7.62 (m, 10H), 7.88–8.20 (m, 4H), 9.08 (s, 1H).MS: *m*/*z* (%) 498.38 (M, 100). Anal. for C_31_H_22_N_4_O_3_ (498.53); Calcd. C, 74.69; H, 4.45; N, 11.24. Found: C, 74.66; H, 4.38; N, 11.29.

*4-(3-(Benzofuran-2-yl)-1-phenyl-1H-pyrazol-4-yl)-2-ethoxy-6-(4-methoxyphenyl)pyridine-3-carbonitrile* (**4l**): yield 2.14 g (41.7%), m.p. 168–170 °C. IR: ν_max_/cm^−1^ 2219 (C≡N), 1589, 1543 (C=N, C=C). ^1^H-NMR (CDCl_3_): δ ^1^H-NMR (CDCl_3_): δ 1.54 (t, *J* = 7 Hz, 3H), 3.83 (s, 3H), 4.67 (q, *J* = 7 Hz, 2H), 6.90 (s, 1H), 7.05 (s, 1H), 7.45–7.52 (m, 9H), 7.81–7.90 (m, 4H), 8.40 (s, 1H). ^13^C-NMR (CDCl_3_): δ 14.7, 55.5, 63.4, 92.6, 105.8, 111.6, 113.4, 114.3, 117.8, 120, 121.4, 123.3, 125.1, 127.8, 128.4, 128.9, 129, 129.7, 139.3, 142.4, 147.1, 149, 154.9, 157.4, 161.7, 164.7. MS: *m*/*z* (%) 512 (M, 22.4). Anal. for C_32_H_24_N_4_O_3_ (512.56); Calcd. C, 74.99; H, 4.72; N, 10.93. Found: C, 74.91; H, 4.71; N, 10.89.

*4-(3-(Benzofuran-2-yl)-1-phenyl-1H-pyrazol-4-yl)-6-(1,2,3,4-tetrahydronaphthalen-6-yl)-2-methoxypyridine-3-carbonitrile* (**4m**): yield 1.78 g (34%), m.p. 255–257 °C. IR: ν_max_/cm^−1^ 2221 (C≡N), 1591, 1555 (C=N, C=C). ^1^H-NMR (CDCl_3_): δ 1.58 (s, 2H), 1.79 (s, 2H), 2.69 (s, 2H), 2.78 (s, 2H), 4.22 (s, 3H), 7.06 (s, 1H), 7.24–7.58 (m, 10H), 7.81–7.83 (m, 4H), 8.43 (s, 1H). ^13^C-NMR (CDCl_3_): δ 23.1, 29.6, 54.6, 92.8, 105.9, 111.6, 114.3, 119.9, 121.4, 123.0, 124.5, 125.1, 127.8, 128.1, 128.4, 128.9, 129.7, 129.9, 137.3, 155.0, 158.2, 165.0. Anal. for C_34_H_26_N_4_O_2_ (522.6); Calcd. C, 78.14; H, 5.01; N, 10.72. Found: C, 73.69; H, 4.14; N, 15.56.

*4-(3-(Benzofuran-2-yl)-1-phenyl-1H-pyrazol-4-yl)-2-ethoxy-6-(1,2,3,4-tetrahydronaphthalen-6-yl)pyridine-3-carbonitrile* (**4n**): Yield 2.16 g (40.3%), m.p. 213–215 °C. IR: ν_max_/cm^−1^ 2213 (C≡N), 1585, 1542 (C=N, C=C). ^1^H-NMR (CDCl_3_): δ 1.54 (t, *J* = 7.7 Hz, 3H), 1.78 (s, 4H), 2.69 (s, 2H), 2.78 (s, 2H), 4.68 (q, *J* = 7.7 Hz, 2H), 7.05 (s, 1H), 7.12–7.56 (m, 11H), 7.81 (d, *J* = 8.5 Hz, 2H), 8.42 (s, 1H). ^13^C-NMR (CDCl_3_): δ 14.7, 23.1, 23.2, 29.6, 29.6 63.4, 92.9, 105.9, 111.6, 114.0, 116.0, 118.0, 120.0, 121.4, 123.3, 124.5, 125.1, 127.8, 128.1, 128.9, 129.7, 129.8, 135.0, 137.8, 140.3, 148.5, 149.5, 155.0, 158.2, 164.8. MS: *m*/*z* (%) 536.31 (M, 100). Anal. for C_35_H_28_N_4_O_2_ (536.62); Calcd. C, 78.34; H, 5.26; N, 10.44. Found: C, 78.52; H, 5.14; N, 10.57.

*4-(3-(Benzofuran-2-yl)-1-phenyl-1H-pyrazol-4-yl)-2-methoxy-6-(1H-pyrrol-2-yl)pyridine-3-carbonitrile* (**4o**): yield 1.86 g (40.6%), m.p. 187–189 °C. IR: ν_max_/cm^−1^ 2218 (C≡N), 1587, 1539 (C=N, C=C). ^1^H-NMR (CDCl_3_): δ 3.97 (s, 3H), 7.06 (s, 1H), 7.29–7.55 (m, 10H), 7.59–7.82 (m, 2H), 8.45 (s, 1H), 9.05 (s, 1H). ^13^C-NMR (CDCl_3_): δ 54.5, 102.0, 106.3, 111.6, 115.4, 117.0, 120.0, 121.7, 123.6, 125.4, 128.2, 128.4, 129.8, 129.9, 138.9, 139.1, 145.7, 148.9, 155.3, 162.4. MS: *m*/*z* (%) 457.25 (M, 1.93). Anal. for C_28_H_19_N_5_O_2_ (457.48); Calcd. C, 73.51; H, 4.19; N, 15.31. Found: C, 73.69; H, 4.14; N, 15.56.

*4-(3-(Benzofuran-2-yl)-1-phenyl-1H-pyrazol-4-yl)-2-ethoxy-6-(1H-pyrrol-2-yl)pyridine-3-carbonitrile* (**4p**): yield 1.84 g (39%), m.p. 208–210 °C. IR: ν_max_/cm^−1^ 2217 (C≡N), 1588, 1538 (C=N, C=C). ^1^H-NMR (CDCl_3_): δ 1.53 (t, *J* = 7.7 Hz, 3H), 4.64 (q, *J* = 7.7 Hz, 2H), 7.05 (s, 1H), 7.20–7.30 (m, 4H), 7.46–7.54 (m, 8H), 7.81 (d, 2H), 8.40 (s, 1H). ^13^C-NMR (CDCl_3_): δ 14.6, 63.8, 93.0, 105.8, 111.6, 112.7, 120.1, 121.4, 123.3, 125.1, 127.1, 127.9, 128.6, 129.0, 129.7, 130.0, 139.3, 142.4, 143.4, 147.2, 148.8, 152.9, 154.9, 164.7. MS: *m*/*z* (%) 471.37 (M, 100). Anal. for C_29_H_21_N_5_O_2_ (471.51); Calcd. C, 73.87; H, 4.49; N, 14.85. Found: C, 73.75; H, 4.43; N, 14.88.

*4-(3-(Benzofuran-2-yl)-1-phenyl-1H-pyrazol-4-yl)-6-(furan-2-yl)-2-methoxypyridine-3-carbonitrile* (**4q**): yield 1.88 g (41%), m.p. 246–248 °C. IR: ν_max_/cm^−1^ 2217 (C≡N), 1588, 1538 (C=N, C=C). ^1^H-NMR (CDCl_3_): δ 4.21 (s, 3H), 6.54 (s, 1H), 7.05 (s, 1H), 7.20–7.53 (m, 10H), 7.80 (d, 2H), 8.35 (s, 1H). ^13^C-NMR (CDCl_3_): δ 54.7, 93.3, 105.7, 111.6, 112.3, 112.6, 117.4, 120.0, 121.4, 123.2, 125.0, 127.9, 128.4, 129.0, 129.7, 139.3, 142.4, 145.1, 147.6, 148.7, 149.3, 152.5, 154.9, 165.1. MS: *m*/*z* (%) 458.21 (M, 100). Anal. for C_28_H_18_N_4_O_3_ (458.47); Calcd. C, 73.35; H, 3.96; N, 12.22. Found: C, 71.22; H, 4.14; N, 11.49.

*4-(3-(Benzofuran-2-yl)-1-phenyl-1H-pyrazol-4-yl)-2-ethoxy-6-(furan-2-yl)pyridine-3-carbonitrile* (**4r**): Yield 2.14 g (45.3%), m.p. 174–176 °C. IR: ν_max_/cm^−1^ 2216 (C≡N), 1590, 1521 (C=N, C=C). ^1^H-NMR (CDCl_3_): δ 1.51 (t, *J* = 7 Hz, 3H), 4.62 (q, *J* = 7 Hz, 2H), 6.53 (s, 1H), 7.00 (s, 1H), 7.15–7.30 (m, 3H), 7.39–7.55 (m, 7H), 7.81 (d, 2H), 8.35 (s, 1H). Anal. for C_29_H_20_N_4_O_3_ (472.49); Calcd. C, 73.72; H, 4.27; N, 11.86. Found: C, 73.65; H, 4.29; N, 11.83.

*4-(3-(Benzofuran-2-yl)-1-phenyl-1H-pyrazol-4-yl)-2-methoxy-6-(thiophen-2-yl)pyridine-3-carbonitrile* (**4s**): yield 1.58 g (33.3%), m.p. 232–234 °C. IR: ν_max_/cm^−1^ 2211 (C≡N), 1629, 1523 (C=N, C=C). ^1^H-NMR (CDCl_3_): δ 4.19 (s, 3H), 7.06 (s, 1H), 7.25–7.52 (m, 11H), 7.81 (d, 2H), 8.40 (s, 1H). ^13^C-NMR (CDCl_3_): δ 54.8, 92.9, 105.8, 111.6, 112.9, 120.1, 121.4, 123.3, 127.2, 127.9, 128.6, 129.1, 129.7, 130.1, 139.3, 142.4, 143.2, 148.8, 153.0, 154.9, 165.0. MS: *m*/*z* (%) 474.18 (M, 100). Anal. for C_28_H_18_N_4_O_2_S (474.53); Calcd. C, 70.87; H, 3.82; N, 11.81. Found: C, 70.79; H, 3.74; N, 11.74.

*4-(3-(Benzofuran-2-yl)-1-phenyl-1H-pyrazol-4-yl)-2-ethoxy-6-(thiophen-2-yl)pyridine-3-carbonitrile* (**4t**): Yield 2.06 g (42.2%), m.p. 202–204 °C. IR: ν_max_/cm^−1^ 2217 (C≡N), 1591, 1546 (C=N, C=C). ^1^H-NMR (CDCl_3_): ^1^H-NMR (CDCl_3_): δ 1.52 (t, *J* = 7 Hz, 3H), 4.63 (q, *J* = 7 Hz, 2H), 6.35 (s, 1H), 6.65 (s, 1H), 7.26–7.52 (m, 3H), 7.64–7.83 (m, 7H), 8.25 (d, *J* = 8 Hz, 2H), 8.36 (s, 1H), 9.65 (s, 1H). ^13^C-NMR (CDCl_3_): δ 14.6. 63.8, 93.0, 105.8, 111.6, 112.7, 115.5, 117.5, 120.1, 121.4, 123.4, 125.1, 127.1, 127.9, 128.6, 129.0, 129.7, 130.0, 139.3, 142.4, 143.4, 147.2, 148.8, 153.0, 155.0, 165.0. MS: *m*/*z* (%) 488.34 (M, 100). Anal. for C_29_H_20_N_4_O_2_S (488.56); Calcd. C, 71.29; H, 4.13; N, 11.47. Found: C, 71.22; H, 4.14; N, 11.49.

*4-(3-(Benzofuran-2-yl)-1-phenyl-1H-pyrazol-4-yl)-2-methoxy-6-(pyridin-2-yl)pyridine-3-carbonitrile* (**4u**): Yield 1.85 g (39.4%), m.p. 275–277 °C. IR: ν_max_/cm^−1^ 2218 (C≡N), 1588, 1545 (C=N, C=C). ^1^H-NMR (DMSO-*d*_6_): δ 4.18 (s, 3H), 7.09 (s, 1H), 7.25–7.62 (m, 10H), 7.98 (d, *J* = 6.7 Hz, 2H), 8.26 (s, 1H), 8.66 (s, 1H), 9.15 (s, 1H).MS: *m*/*z* (%) 469.39 (M, 100). Anal. for C_29_H_19_N_5_O_2_ (469.49); Calcd. C, 74.19; H, 4.08; N, 14.92. Found: C, 74.24; H, 4.15; N, 14.86.

*4-(3-(Benzofuran-2-yl)-1-phenyl-1H-pyrazol-4-yl)-2-ethoxy-6-(pyridin-2-yl)pyridine-3-carbonitrile* (**4v**): yield 1.75 g (36.2%), m.p. 204–206 °C. IR: ν_max_/cm^−1^ 2219 (C≡N), 1589, 1543 (C=N, C=C). ^1^H-NMR (CDCl_3_): δ 1.55 (t, *J* = 6.7 Hz, 3H), 4.69 (q, *J* = 6.7 Hz, 2H), 7.03 (s, 1H), 7.19–7.53 (m, 13H), 7.82 (d, *J* = 8.6 Hz, 2H), 8.27–8.35 (m, 2H), 8.65 (s, 1H). ^13^C-NMR (CDCl_3_): δ 14.6, 63.7, 95.8, 104.9, 105.5, 111.6, 119.8, 120.0, 121.3, 123.1, 124.8, 128.9, 129.7, 137.0, 139.4, 142.5, 142.6, 148.1, 149.6, 154.3, 156.5, 164.6. MS: *m*/*z* (%) 483.25 (M, 100). Anal. for C_30_H_21_N_5_O_2_ (483.52); Calcd. C, 74.52; H, 4.38; N, 14.48. Found: C, 74.36; H, 4.24; N, 11.56.

*4-(3-(Benzofuran-2-yl)-1-phenyl-1H-pyrazol-4-yl)-2-methoxy-6-(1-methyl-1H-benzo[d]imidazole-2-yl)pyridine-3-carbonitrile* (**4w**): yield 2.05 g (39.2%), m.p. 295–297 °C. IR: ν_max_/cm^−1^ 2214 (C≡N), 1586, 1538 (C=N, C=C). ^1^H-NMR (CDCl_3_): δ 4.18 (s, 3H), 4.35 (s, 3H), 7.14 (s, 1H), 7.27–8.01 (m, 13H), 8.26 (s, 1H), 9.16 (s, 1H).MS: *m*/*z* (%) 522.1 (M, 20.5). Anal. for C_32_H_22_N_6_O_2_ (522.56); Calcd. C, 73.55; H, 4.24; N, 16.08. Found: C, 73.49; H, 4.22; N, 16.13.

*4-(3-(Benzofuran-2-yl)-1-phenyl-1H-pyrazol-4-yl)-2-ethoxy-6-(1-methyl-1H-benzo[d]-imidazol-2-yl)pyridine-3-carbonitrile* (**4x**): yield 2.12 g (39.5%), m.p. 247–249 °C. IR: ν_max_/cm^−1^ 2218 (C≡N), 1587, 1539 (C=N, C=C). ^1^H-NMR (CDCl_3_): δ 1.56 (t, *J* = 7 Hz, 3H), 4.36 (q, *J* = 7 Hz, 2H), 4.64 (s, 3H), 7.1 (s, 1H), 7.20–7.53 (m, 11H, Ar–H), 7.77, 7.83 (dd, 2H, *J* = 7.65, *J* = 7.65, Ar–H), 8.33 (s, 1H), 8.39 (s, 1H). ^13^C-NMR (CDCl_3_): δ 14.6, 33.2, 64.1, 95.9, 105.4, 110.1, 111.7, 118.7, 120.0, 120.6, 121.4, 123.1, 123.3, 124.4, 124.8, 127.8, 128.9, 129.7, 137.5, 139.3, 142.5, 142.7, 148.1, 148.3, 148.9, 150.7, 155.0, 164.3. MS: *m*/*z* (%) 536.44 (M, 100). Anal. for C_33_H_24_N_6_O_2_ (536.58); Calcd. C, 73.87; H, 4.51; N, 15.66. Found: C, 73.91; H, 4.45; N, 15.58.

### 3.2. Vasodilation Activity Screening

The vasodilation activity screening procedures were carried out according to the standard reported techniques [35] by testing the effects of the synthesized 2-alkoxy-4-aryl-6-(benzofuran-2-yl)-3-pyridinecarbonitriles **4a**–**x** on isolated thoracic aortic rings of male Wistar rats (250–350 g). After light ether anesthesia, the rats were sacrificed by cervical dislocation. The aortae were immediately excised, freed of extraneous tissues and prepared for isometric tension recording. Aorta was cut into (3–5 mm width) rings and each ring was placed in a vertical chamber “10 mL jacketed automatic multi-chamber organ bath system (Model no. ML870B6/C, Panlab, Spain)” filled with Krebs solution composed of (in mM): NaCl, 118.0; KCl, 4.7; NaHCO_3_, 25.0; CaCl_2_, 1.8; NaH_2_PO_4_, 1.2; MgSO_4_, 1.2; glucose, 11.0 and oxygenated with carbogen gas (95% O_2_/5% CO_2_) at 37 ± 0.5 °C. Each aortic ring was mounted between two stainless steel hooks passed through its lumen. The lower hook was fixed between two plates, while the upper one was attached to a force displacement transducer (Model no. MLT0201, Panlab, Spain) connected to an amplifier (PowerLab, AD Instruments Pty., Ltd. Victoria, Australia), which is connected to a computer. The chart for Windows (v 3.4) software was used to record and elaborate data. Preparations were stabilized under 2 g resting tension during 2 h and then the contracture response to norepinephrine hydrochloride (10^−6^ M) was measured before and after exposure to increasing concentrations of the tested synthesized compounds. The tested compounds were dissolved in dimethylsulfoxide (DMSO) as stock solution (10 mL of 0.005 M). Control experiments were performed in the presence of DMSO alone, at the same concentrations as those used with the derivatives tested, which demonstrated that the solvent did not affect the contractile response of isolated aorta. The observed vasodilation activity screening data are reported and the potency (IC_50_, concentration necessary for 50% reduction of maximal norepinephrine hydrochloride induced contracture) was calculated in three successful replicates and the observed vasodilation activity data expressed as IC_50_ has determined mathematically from the dose response curve of each tested compound (Table 1), (Figures S1 and S2 of Supplementary Materials) and the potency (IC_50_, concentration necessary for 50% reduction of maximal norepinephrine hydrochloride-induced contracture) was determined. This experiment was carried out in according to recommendations in the Guide for the Care and Use of Laboratory Animals of the National Institutes of Health (NIH publication No. 85–23, revised 1996) and under regulations of the Animal Care and Use of National Research Centre in Egypt.

#### 2D-QSAR Study

The QSAR study was undertaken using comprehensive descriptors for structural and statistical analysis (CODESSA PRO) software employing the synthesized compounds **4a**–**x** of the present study (Table 3). Geometry of the compounds was optimized using molecular mechanics force field (MM^+^) followed by the semiempirical AM1 method implemented in the HyperChem 8.0 package. The structures were fully optimized without fixing any parameters, thus bringing all geometric variables to their equilibrium values. The energy minimization protocol employed the Polake–Ribiere conjugated gradient algorithm. Convergence to a local minimum was achieved when the energy gradient was ≤0.01 kcal/mol. The Restricted Hartree–Fock (RHF) method was used in spin-pairing for the two semiempirical tools [39,40,41,42,43,44]. The resulting output files were exported to CODESSA PRO that includes MOPAC capability for final geometry optimization. CODESSA PRO calculated 797 molecular descriptors including constitutional, topological, geometrical, charge-related, semiempirical, thermodynamic, molecular-type, atomic-type and bond-type descriptors for the exported 24 bioactive benzofuran-based hybrids **4a**–**x**, which were used in the present study. Different mathematical transformations of the experimentally observed property/activity (IC_50_, µM, which is the concentration necessary for 50% reduction of maximal norepinephrine hydrochloride induced contracture) of the training set compounds were utilized for the present QSAR modeling determination including property (IC_50_, µM), 1/property, log(property) and 1/log(property) values in searching for the best QSAR models. Best multilinear regression (BMLR) was utilized, which is a stepwise search for the best n-parameter regression equations (where, n stands for the number of descriptors used), based on the highest *R*^2^ (squared correlation coefficient), *R*^2^_cv_OO (squared cross-validation “leave one-out, LOO” coefficient), *R*^2^_cv_MO (squared cross-validation “leave many-out, LMO” coefficient), *F* (Fisher statistical significance criteria) values, and *s*^2^ (standard deviation). The QSAR models, with up to four descriptor models describing the bioactivity of the vasodilatory active agents were generated (obeying the thumb rule of 6:1, which is the ratio between the data points and the number of QSAR descriptor models). Statistical characteristics of the QSAR models are presented in Table 2. The established QSAR model is statistically significant. The descriptors are sorted in descending order of the respective values of the Student’s *t*-criterion, which is a widely accepted measure of statistical significance of individual parameters in multiple linear regressions. Figure 2 exhibits the QSAR multilinear model plot of correlation representing the observed vs. predicted 1/IC_50_ values for vasodilatory active agents. The scattered plots are uniformly distributed, covering ranges, Observed 0.00221–0.00448; Predicted 0.00255–0.004411/IC_50_ units.

### 3.3. Toxicological Bioassay

Toxicological bioassay of the most promising vasodilatory active compounds (**4a**, **c**, **e**, **f**, **g**, **l**, **m**, **r**, **s**, **t**, **w** and **4x**) was determined using the standard reported method in mice [45]. Albino mice weighing 25–30 g were divided in 13 groups of 6 mice each. Administrations of the tested compounds dissolved in saline solution (0.9%) by the aid of few drops of Tween 80 were given intraperitoneally in 1000 mg kg^−1^ (mouse body weight). The control group was given saline solution only with few drops of Tween 80. The toxic symptoms and mortality rates were recorded 24 h post-administration in each group.

## 4. Conclusions

The required 2-alkyloxy-pyridine-3-carbonitrile hybrids (**4a**–**x**) were designed and synthesized via the condensation reaction of aromatic ketones **3a**‒**l** with 2-((3-(benzofuran-2-yl)-1-phenyl-1*H*-pyrazol-4-yl)methylene)malononitrile (**2**) in the presence of sufficient amount of sodium alkoxide in the corresponding alcohol. The compounds have evaluated for their vasodilation activity adopting the standard technique “using isolated thoracic aortic rings of rats precontracted with norepinephrine hydrochloride”. Some compounds revealed a noteworthy activity, with compounds **4w**, **4e**, **4r**, **4s**, **4f** and **4g** believed to be the most active hits in this study. “IC_50_, concentration necessary for 50% reduction of maximal norepinephrine hydrochloride induced contracture = 223, 253, 254, 268, 267 and 275 µM, respectively”, compared with amiodarone hydrochloride, the reference standard used (IC_50_ = 300 μM). The CODESSA PRO program was utilized to achieve a statistically significant 2D-QSAR model describing the bioactivity of the newly synthesized analogues **4a**–**x**, and afforded an excellent predictive and statistically vital four-crucial 4 descriptor model (*R*^2^ = 0.816, *R*^2^_observed_ = 0.731, *R*^2^_pridicted_ = 0.772). It is obvious that the 2D-QSAR study supported the attained model, so the applicability of benzofuran-based hybrids incorporating the 3-pyridinecarbonitrile function have potential to be developed into vasorelaxant active agents.

## Figures and Tables

**Table:** 

**3a**; R = Ph	**4a**; R = Ph
**3b**; R = 4-ClC_6_H_4_	**4b**; R = Ph
**3c**; R = 4-BrC_6_H_4_	**4c**; R = 4-ClC_6_H_4_
**3d**; R = 4-FC_6_H_4_	**4d**; R = 4-ClC_6_H_4_
**3e**; R = 4-H_3_CC_6_H_4_	**4e**; R = 4-BrC_6_H_4_
**3f**; R = 4-H_3_COC_6_H_4_	**4f**; R = 4-BrC_6_H_4_
**3g**; R = 1,2,3,4-Tetrahydronaphthalen-6-yl	**4g**; R = 4-FC_6_H_4_
**3h**; R = 2-Pyrrolyl	**4h**; R = 4-FC_6_H_4_
**3i**; R = 2-Furanyl	**4i**; R = 4-H_3_CC_6_H_4_
**3j**; R = 2-Thienyl	**4j**; R = 4-H_3_CC_6_H_4_
**3k**; R = 2-Pyridinyl	**4k**; R = 4-H_3_COC_6_H_4_
**3l**; R = 1-Methyl-1*H*-benzo[*d*]imidazol-2-yl	**4l**; R = 4-H_3_COC_6_H_4_
	**4m**; R = 1,2,3,4-Tetrahydronaphthalen-6-yl, R′ = Me
	**4n**; R = 1,2,3,4-Tetrahydronaphthalen-6-yl, R′ = Et
	**4o**; R = 2-Pyrrolyl, R′ = Me
	**4p**; R = 2- Pyrrolyl, R′ = Et
	**4q**; R = 2-Furanyl, R′ = Me
	**4r**; R = 2-Furanyl, R′ = Et
	**4s**; R = 2-Thienyl, R′ = Me
	**4t**; R = 2-Thienyl, R′ = Et
	**4u**; R = 2-Pyridinyl, R′ = Me
	**4v**; R = 2-Pyridinyl, R′ = Et
	**4w**;R = 1-Methyl-1*H*-benzo[*d*]imidazol-2-yl, R′ = Me
	**4x**; R = 1-Methyl-1*H*-benzo[*d*]imidazol-2-yl, R′ = Et

*Reagents and Conditions*: (**i**) CNCH_2_CN/Ethanol/r.t./6 h, 75%; (**ii**) alcohol/Na/r.t./33.3–45.3%.

**Table 1:** Vasodilatory activity IC_50_ (mM) in rat thoracic aortic rings.

Entry	Compound	R	R′	Potency (IC_50_), mM
1	**4a**	Ph	Me	0.281
2	**4b**	Ph	Et	0.343
3	**4c**	4-ClC_6_H_4_	Me	0.295
4	**4d**	4-ClC_6_H_4_	Et	0.397
5	**4e**	4-BrC_6_H_4_	Me	0.253
6	**4f**	4-BrC_6_H_4_	Et	0.267
7	**4g**	4-FC_6_H_4_	Me	0.275
8	**4h**	4-FC_6_H_4_	Et	0.330
9	**4i**	4-H_3_CC_6_H_4_	Me	0.330
10	**4j**	4-H_3_CC_6_H_4_	Et	0.452
11	**4k**	4-H_3_COC_6_H_4_	Me	0.322
12	**41**	4-H_3_COC_6_H_4_	Et	0.291
13	**4m**	1,2,3,4-Tetrahydronaphthalen-6-yl	Me	0.286
14	**4n**	1,2,3,4-Tetrahydronaphthalen-6-yl	Et	0.337
15	**4o**	2-Pyrrolyl	Me	0.356
16	**4p**	2-Pyrrolyl	Et	0.400
17	**4q**	2-Furanyl	Me	0.321
18	**4r**	2-Furanyl	Et	0.254
19	**4s**	2-Thienyl	Me	0.268
20	**4t**	2-Thienyl	Et	0.298
21	**4u**	2-Pyridinyl	Me	0.333
22	**4v**	2-Pyridinyl	Et	0.370
23	**4w**	1-Methyl-1*H*-benzo[*d*]imidazol-2-yl	Me	0.223
24	**4x**	1-Methyl-1*H*-benzo[*d*]imidazol-2-yl	Et	0.299
25	Amiodarone.HCl	-	-	0.300

**Table 2:** Descriptors of the BMLR-QSAR model for the vasodilatory active compounds.

Entry	ID	Coefficient	*s*	*t*	Descriptor
*N* = 24, *n*= 4, *R*^2^ = 0.816, *R*^2^_cv_OO = 0.731, *R*^2^_cv_MO = 0.772, *F* = 21.103, *s*^2^ = 6.191 × 10^−8^					
1	0	1.464	0.244	6.009	Intercept
2	*D*_1_	0.0004	4.751 × 10^−5^	7.789	Max. e–e repulsion for bond C–O
3	*D*_2_	−7.1825 × 10^−6^	1.975 × 10^−6^	−3.637	*WNSA*-1 Weighted *PNSA* (PNSA1*TMSA/1000) (MOPAC PC)
4	*D*_3_	−0.255	0.045	−5.670	*FHACA* Fractional *HACA* (*HACA1*/*TMSA*) (MOPAC PC)
5	*D*_4_	−0.0043	0.001	−6.241	Max. e–n attraction for bond C–N
1/IC_50_ (μM) = 1.464 + (0.0004 × *D*_1_) − [(7.1825 × 10^−6^) × *D*_2_] − (0.255 × *D*_3_) − (0.0043 × *D*_3_)					

**Table 3:** Observed and estimated/predicated values of the vasodilatory active compounds according to the BMLR-QSAR model.

Entry	Compd.	R	R′	Observed IC_50_, μM	Estimated IC_50_, μM	Error
1	**4a**	Ph	Me	281	276	5
2	**4b**	Ph	Et	343	363	−20
3	**4c**	4-ClC_6_H_4_	Me	295	272	23
4	**4d**	4-ClC_6_H_4_	Et	397	392	5
5	**4e**	4-BrC_6_H_4_	Me	253	253	0
6	**4f**	4-BrC_6_H_4_	Et	267	307	−40
7	**4g**	4-FC_6_H_4_	Me	275	303	−28
8	**4h**	4-FC_6_H_4_	Et	330	354	−24
9	**4i**	4-H_3_CC_6_H_4_	Me	330	299	31
10	**4j**	4-H_3_CC_6_H_4_	Et	452	369	83
11	**4k**	4-H_3_COC_6_H_4_	Me	322	337	−15
12	**41**	4-H_3_COC_6_H_4_	Et	291	307	−16
13	**4m**	1,2,3,4-Tetrahydronaphthalen-6-yl	Me	286	265	21
14	**4n**	1,2,3,4-Tetrahydronaphthalen-6-yl	Et	337	344	−7
15	**4o**	2-Pyrrolyl	Me	268	286	−18
16	**4p**	2-Pyrrolyl	Et	298	311	−13
17	**4q**	2-Furanyl	Me	321	310	11
18	**4r**	2-Furanyl	Et	254	257	−3
19	**4s**	2-Thienyl	Me	356	328	28
20	**4t**	2-Thienyl	Et	400	386	14
21	**4u**	2-Pyridinyl	Me	333	336	−3
22	**4v**	2-Pyridinyl	Et	370	369	1
23	**4w**	1-Methyl-1*H*-benzo[*d*]imidazol-2-yl	Me	223	227	−4
24	**4x**	1-Methyl-1*H*-benzo[*d*]imidazol-2-yl	Et	299	285	14

## Supplementary Materials

Supplementary materials are available online.
