# Supplementary material for: Synthesis and 2D-QSAR Study of Active Benzofuran-Based Vasodilators
Source: Molecules. 2017 Oct 26;22(11):1820. doi: 10.3390/molecules22111820 (PMC6150240; doi:10.3390/molecules22111820)
Supplement: Supplementary file 1 [file molecules-22-01820-s001.pdf]

# Supplementary material

## Figure Captions

**Table S1.** Molecular descriptor values of the BMLR-QSAR model for the vasodilatory active agents.

**Figure S1.** Effect of synthesized compounds and the standard reference (Amiodarone hydrochloride) on contracture induced by norepinephrine hydrochloride (NE.HCl) in rat thoracic aortic rings.

**Figure S2.** Potency ($IC_{50}$, mM) of the tested compounds on contracture induced by norepinephrine hydrochloride in rat thoracic aortic rings compared with (Amiodarone hydrochloride) used as a reference standard.

**Figure S3.** $^{1}$H NMR spectrum of **4a**.

**Figure S4.** $^{13}$C NMR spectrum of **4a**.

**Figure S5.** $^{1}$H NMR spectrum of **4b**.

**Figure S6.** $^{13}$C NMR spectrum of **4b**.

**Figure S7.** $^{1}$H NMR spectrum of **4c**.

**Figure S8**. $^{1}$H NMR spectrum of **4d**.

**Figure S9.** $^{13}$C NMR spectrum of **4d**.

**Figure S10.** $^{1}$H NMR spectrum of **4e**.

**Figure S11.** $^{1}$H NMR spectrum of **4f**.

**Figure S12.** $^{13}$C NMR spectrum of **4f**.

**Figure S13.** $^{1}$H NMR spectrum of **4g**.

**Figure S14.** $^{1}$H NMR spectrum of **4h**.

**Figure S15.** $^{13}$C NMR spectrum of **4h**.

**Figure S16.** $^{1}$H NMR spectrum of **4i**.

**Figure S17.** $^{13}$C NMR spectrum of **4i**.

**Figure S18.** $^{1}$H NMR spectrum of **4j**.

**Figure S19.** $^{13}$C NMR spectrum of **4j**.

**Figure S20.** $^{1}$H NMR spectrum of **4k**.

**Figure S21.** $^{1}$H NMR spectrum of **4l**.

**Figure S22.** $^{13}$C NMR spectrum of **4l**.

**Figure S23.** $^{1}$H NMR spectrum of **4m**.

**Figure S24.** $^{13}$C NMR spectrum of **4m**.

**Figure S25.** $^{1}$H NMR spectrum of **4n**.

**Figure S26.** $^{13}$C NMR spectrum of **4n**.

**Figure S27.** $^{1}$H NMR spectrum of **4m**.

**Figure S28.** $^{1}$H NMR spectrum of **4o**.

**Figure S29.** $^{1}$H NMR spectrum of **4p**.

**Figure S30.** $^{13}$C NMR spectrum of **4p**.

**Figure S31.** $^{1}$H NMR spectrum of **4q**.

**Figure S32.** $^{13}$C NMR spectrum of **4q**.

**Figure S33.** $^{1}$H NMR spectrum of **4r**.

**Figure S34.** $^{1}$H NMR spectrum of **4s**.

**Figure S35.** $^{13}$C NMR spectrum of **4s**.

**Figure S36.** $^{1}$H NMR spectrum of **4t**.

**Figure S37.** $^{1}$H NMR spectrum of **4u**.

**Figure S38.** $^{1}$H NMR spectrum of **4v**.

**Figure S39.** $^{13}$C NMR spectrum of **4v**.

**Figure S40.** $^{1}$H NMR spectrum of **4w**.

**Figure S41.** $^{1}$H NMR spectrum of **4x**.

**Figure S42.** $^{13}$C NMR spectrum of **4x**.

**Table S1.** Molecular descriptor values of the BMLR-QSAR model for the vasodilatory active agents.

| Entry | Compd. | Descriptors* | | | |
|---|---|---|---|---|---|
| | | $D_1$ | $D_2$ | $D_3$ | $D_4$ |
| 1 | **4a** | 196.7354 | 294.6783 | 0.00796 | 350.9802 |
| 2 | **4b** | 193.9982 | 266.2766 | 0.00769 | 351.0109 |
| 3 | **4c** | 196.7631 | 318.9088 | 0.00693 | 350.993 |
| 4 | **4d** | 193.9671 | 364.9057 | 0.00634 | 350.9712 |
| 5 | **4e** | 196.7521 | 283.3206 | 0.00708 | 350.9765 |
| 6 | **4f** | 193.9576 | 280.1435 | 0.00621 | 350.9558 |
| 7 | **4g** | 196.7126 | 331.4414 | 0.00822 | 350.9776 |
| 8 | **4h** | 193.5552 | 303.7959 | 0.00584 | 351.0034 |
| 9 | **4i** | 196.7445 | 309.5561 | 0.00817 | 351.0101 |
| 10 | **4j** | 193.5273 | 284.8733 | 0.00679 | 351.0018 |
| 11 | **4k** | 198.4084 | 301.8383 | 0.01218 | 351.0151 |
| 12 | **4l** | 198.3929 | 315.658 | 0.01065 | 351.0124 |
| 13 | **4m** | 196.8043 | 256.5694 | 0.00756 | 351.0373 |
| 14 | **4n** | 194.0101 | 302.4001 | 0.00608 | 351.0104 |
| 15 | **4o** | 196.8059 | 261.9922 | 0.01086 | 351.0023 |
| 16 | **4p** | 194.0591 | 256.2957 | 0.00759 | 351.0759 |
| 17 | **4q** | 196.6616 | 274.1543 | 0.01027 | 350.9653 |
| 18 | **4r** | 196.5003 | 255.6172 | 0.00738 | 350.9988 |
| 19 | **4s** | 196.7666 | 255.4456 | 0.00916 | 351.0069 |
| 20 | **4t** | 194.0979 | 236.8933 | 0.00633 | 351.0407 |
| 21 | **4u** | 196.6594 | 296.6829 | 0.01042 | 350.9755 |
| 22 | **4v** | 194.0351 | 271.8696 | 0.00829 | 350.979 |
| 23 | **4w** | 196.657 | 361.9157 | 0.00842 | 350.6562 |
| 24 | **4x** | 193.4009 | 352.4049 | 0.00726 | 350.6691 |

*$D_1$ = Max. e-e repulsion for bond C-O, $D_2$ = WNSA-1 Weighted PNSA (PNSA1*TMSA/1000) (MOPAC PC), $D_3$ = FHACA Fractional HACA (HACA/TMSA) (MOPAC PC), $D_4$ = Max. e-n attraction for bond C-N.

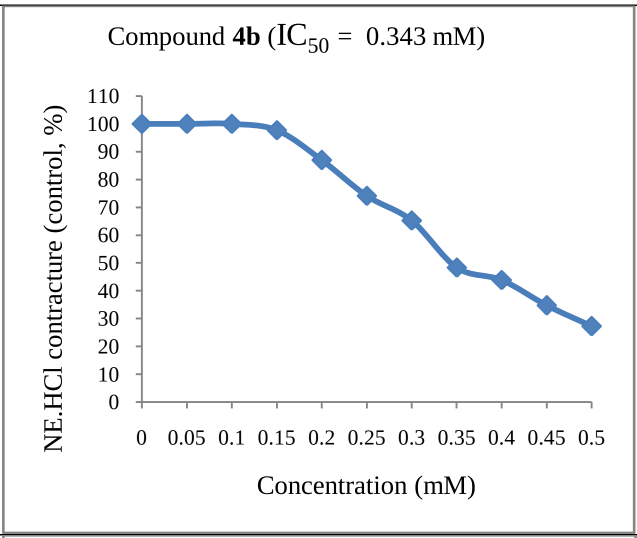
Compound 4b (IC50 = 0.343 mM)
NE.HCl contracture (control, %)
110
100
90
80
70
60
50
40
30
20
10
0
0 0.05 0.1 0.15 0.2 0.25 0.3 0.35 0.4 0.45 0.5
Concentration (mM)

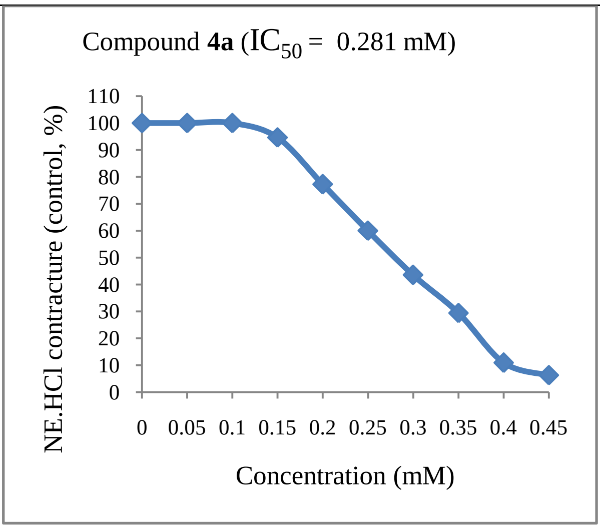
Compound 4a (IC50 = 0.281 mM)
NE.HCl contracture (control, %)
110
100
90
80
70
60
50
40
30
20
10
0
0 0.05 0.1 0.15 0.2 0.25 0.3 0.35 0.4 0.45
Concentration (mM)

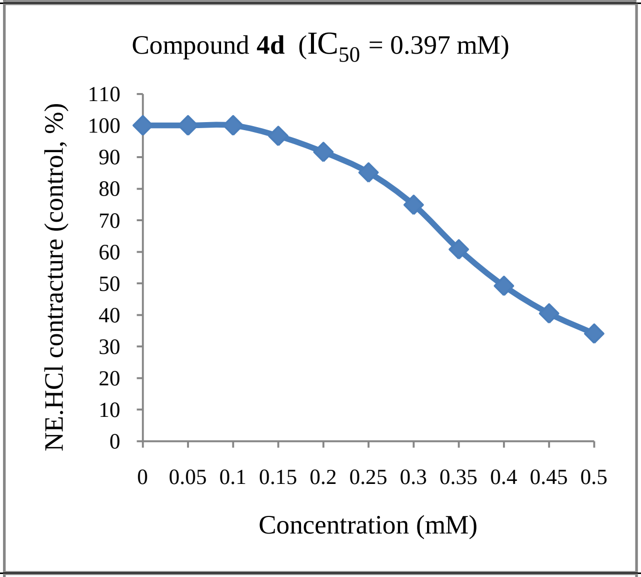
Compound 4d (IC50 = 0.397 mM)
NE.HCl contracture (control, %)
110
100
90
80
70
60
50
40
30
20
10
0
0 0.05 0.1 0.15 0.2 0.25 0.3 0.35 0.4 0.45 0.5
Concentration (mM)

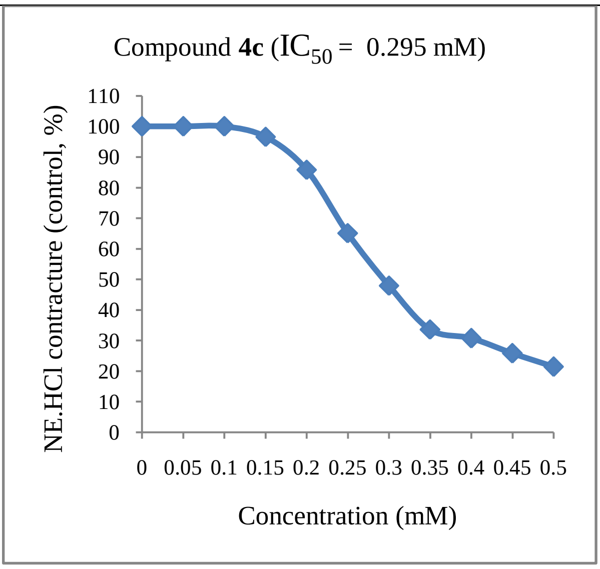
Compound 4c (IC50 = 0.295 mM)
NE.HCl contracture (control, %)
110
100
90
80
70
60
50
40
30
20
10
0
0 0.05 0.1 0.15 0.2 0.25 0.3 0.35 0.4 0.45 0.5
Concentration (mM)

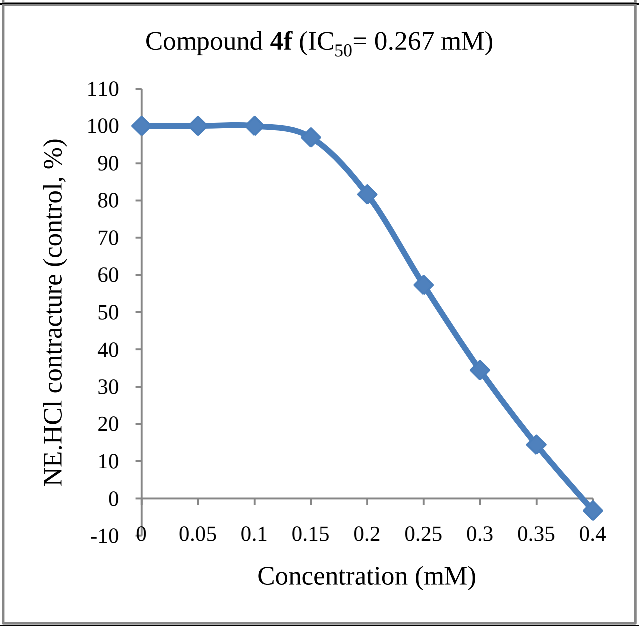
Compound 4f (IC50= 0.267 mM)
NE.HCl contracture (control, %)
110
100
90
80
70
60
50
40
30
20
10
0
-10
0 0.05 0.1 0.15 0.2 0.25 0.3 0.35 0.4
Concentration (mM)

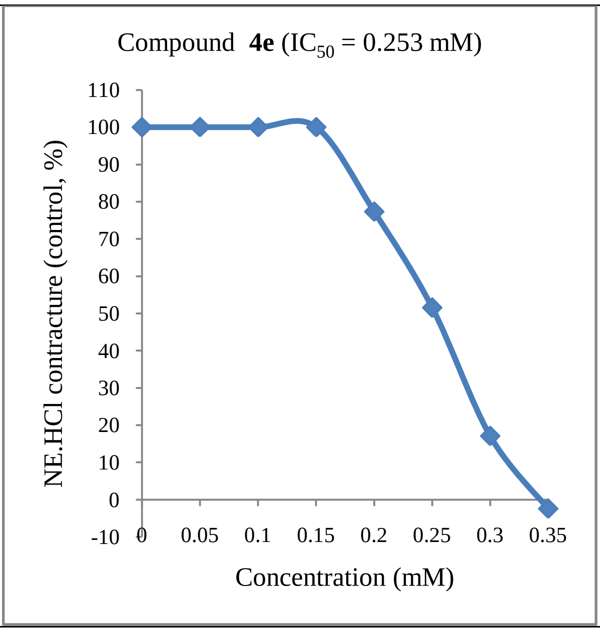
Compound 4e (IC50 = 0.253 mM)
NE.HCl contracture (control, %)
110
100
90
80
70
60
50
40
30
20
10
0
-10
0 0.05 0.1 0.15 0.2 0.25 0.3 0.35
Concentration (mM)

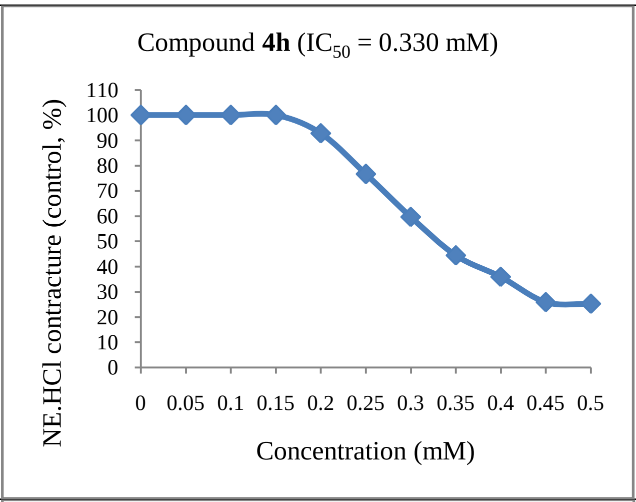
Compound **4h** ($IC_{50}$ = 0.330 mM)
NE.HCl contracture (control, %)
110 100 90 80 70 60 50 40 30 20 10 0
0 0.05 0.1 0.15 0.2 0.25 0.3 0.35 0.4 0.45 0.5
Concentration (mM)

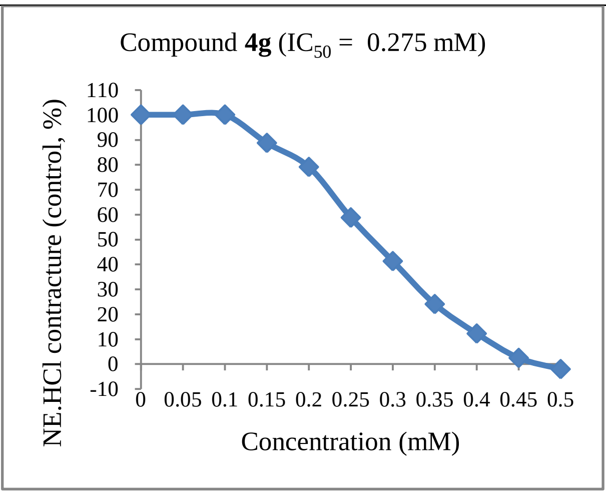
Compound **4g** ($IC_{50}$ = 0.275 mM)
NE.HCl contracture (control, %)
110 100 90 80 70 60 50 40 30 20 10 0 -10
0 0.05 0.1 0.15 0.2 0.25 0.3 0.35 0.4 0.45 0.5
Concentration (mM)

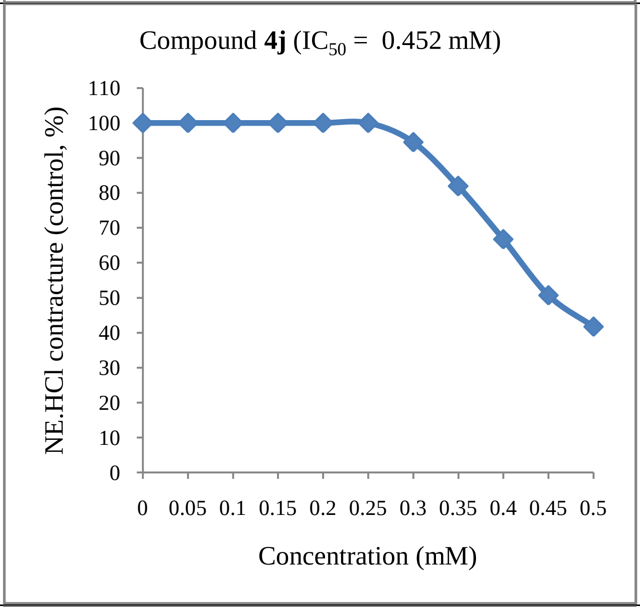
Compound **4j** ($IC_{50}$ = 0.452 mM)
NE.HCl contracture (control, %)
110 100 90 80 70 60 50 40 30 20 10 0
0 0.05 0.1 0.15 0.2 0.25 0.3 0.35 0.4 0.45 0.5
Concentration (mM)

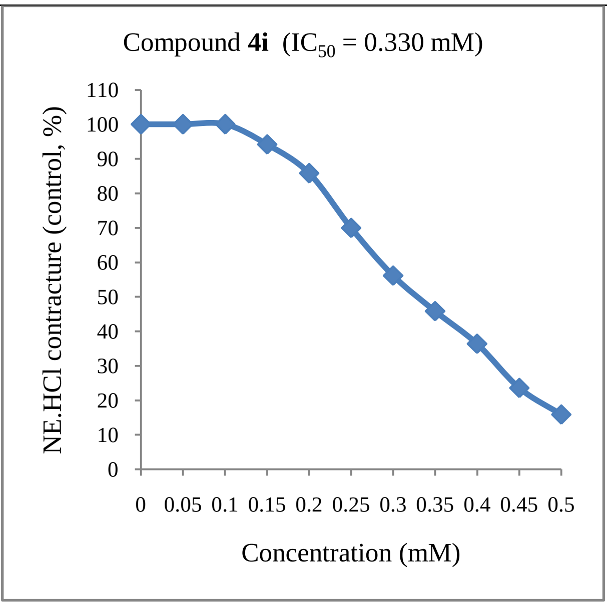
Compound **4i** ($IC_{50}$ = 0.330 mM)
NE.HCl contracture (control, %)
110 100 90 80 70 60 50 40 30 20 10 0
0 0.05 0.1 0.15 0.2 0.25 0.3 0.35 0.4 0.45 0.5
Concentration (mM)

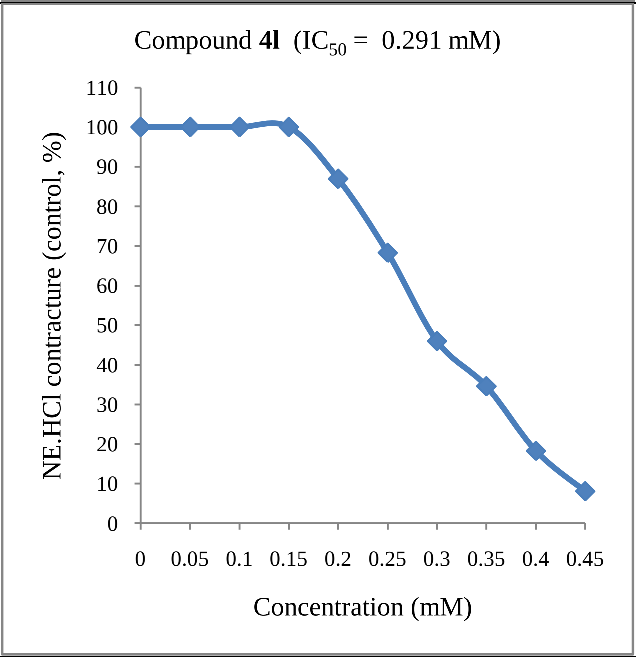
Compound **4l** ($IC_{50}$ = 0.291 mM)
NE.HCl contracture (control, %)
110 100 90 80 70 60 50 40 30 20 10 0
0 0.05 0.1 0.15 0.2 0.25 0.3 0.35 0.4 0.45
Concentration (mM)

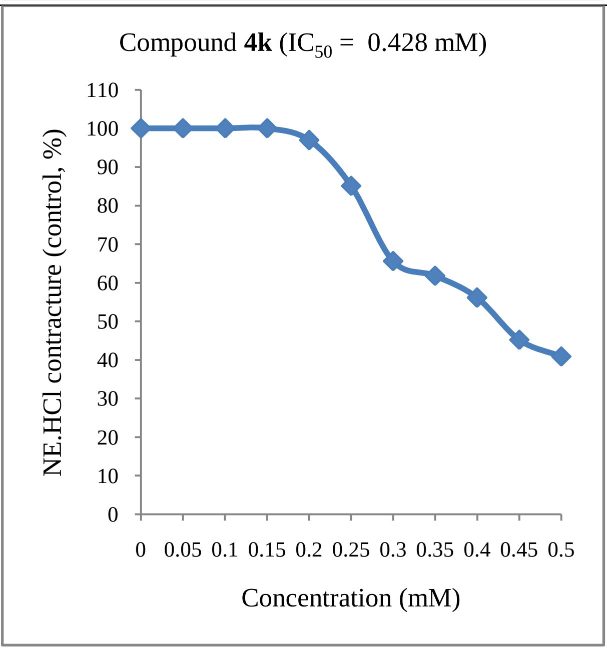
Compound **4k** ($IC_{50}$ = 0.428 mM)
NE.HCl contracture (control, %)
110 100 90 80 70 60 50 40 30 20 10 0
0 0.05 0.1 0.15 0.2 0.25 0.3 0.35 0.4 0.45 0.5
Concentration (mM)

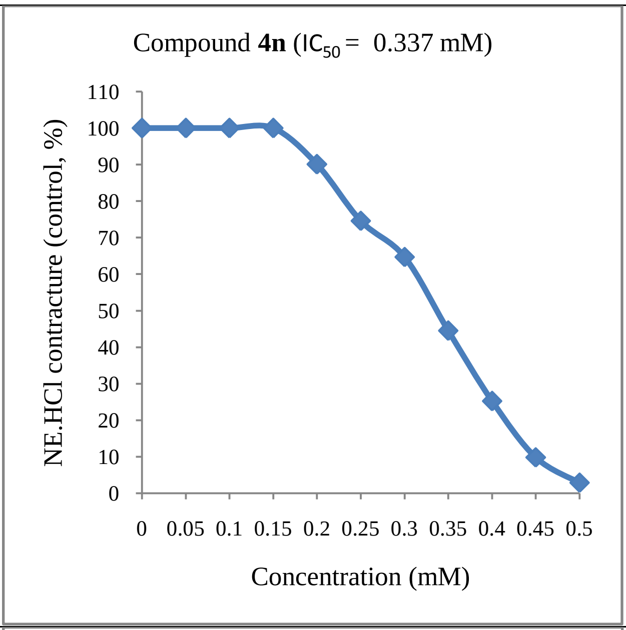
Compound 4n (IC50 = 0.337 mM)
NE.HCl contracture (control, %)
110 100 90 80 70 60 50 40 30 20 10 0
0 0.05 0.1 0.15 0.2 0.25 0.3 0.35 0.4 0.45 0.5
Concentration (mM)

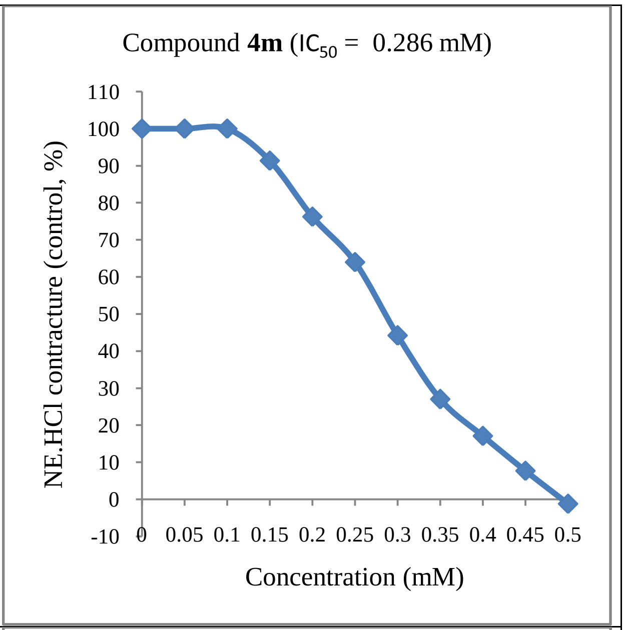
Compound 4m (IC50 = 0.286 mM)
NE.HCl contracture (control, %)
110 100 90 80 70 60 50 40 30 20 10 0 -10
0 0.05 0.1 0.15 0.2 0.25 0.3 0.35 0.4 0.45 0.5
Concentration (mM)

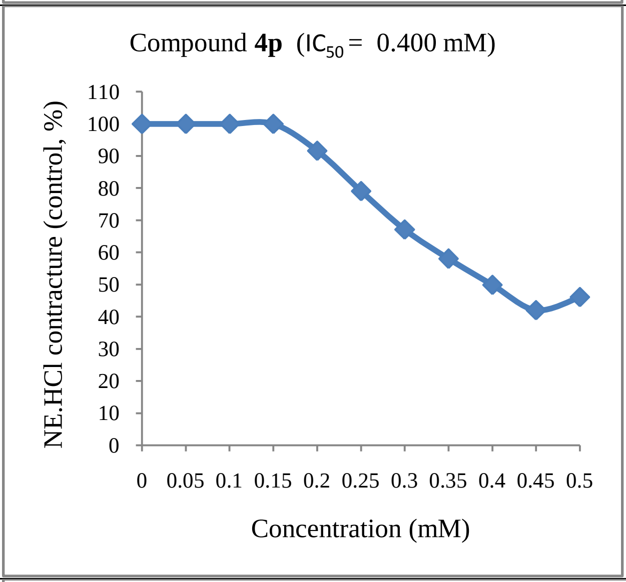
Compound 4p (IC50 = 0.400 mM)
NE.HCl contracture (control, %)
110 100 90 80 70 60 50 40 30 20 10 0
0 0.05 0.1 0.15 0.2 0.25 0.3 0.35 0.4 0.45 0.5
Concentration (mM)

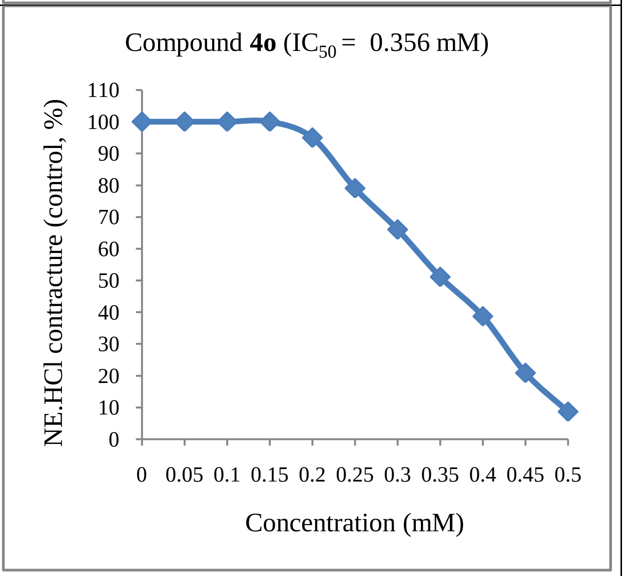
Compound 4o (IC50 = 0.356 mM)
NE.HCl contracture (control, %)
110 100 90 80 70 60 50 40 30 20 10 0
0 0.05 0.1 0.15 0.2 0.25 0.3 0.35 0.4 0.45 0.5
Concentration (mM)

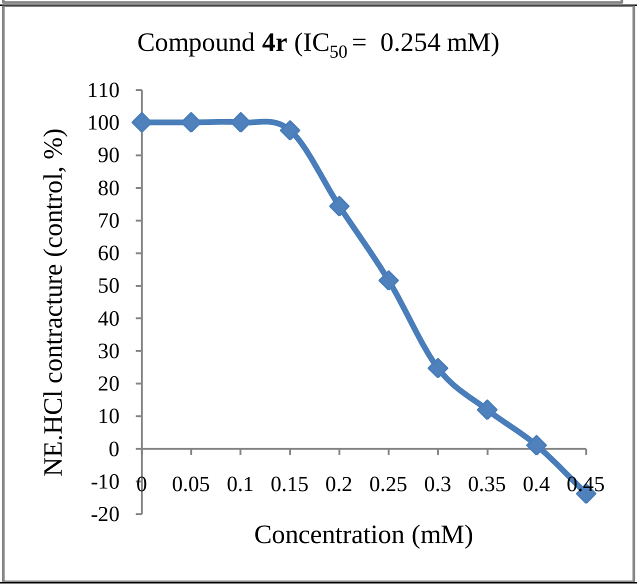
Compound 4r (IC50 = 0.254 mM)
NE.HCl contracture (control, %)
110 100 90 80 70 60 50 40 30 20 10 0 -10 -20
0 0.05 0.1 0.15 0.2 0.25 0.3 0.35 0.4 0.45
Concentration (mM)

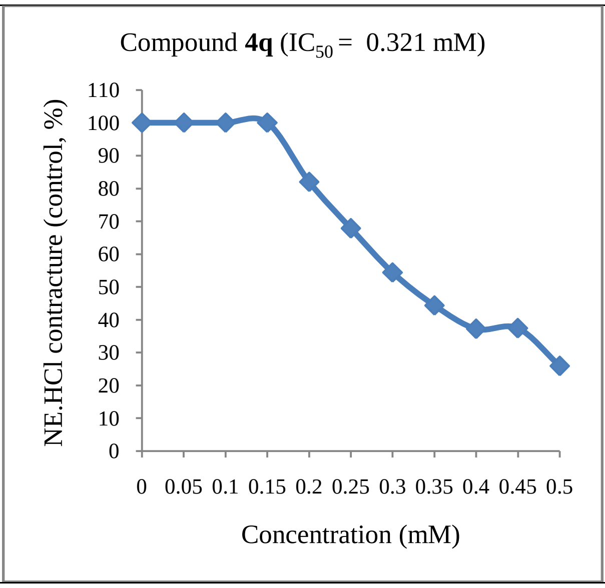
Compound 4q (IC50 = 0.321 mM)
NE.HCl contracture (control, %)
110 100 90 80 70 60 50 40 30 20 10 0
0 0.05 0.1 0.15 0.2 0.25 0.3 0.35 0.4 0.45 0.5
Concentration (mM)

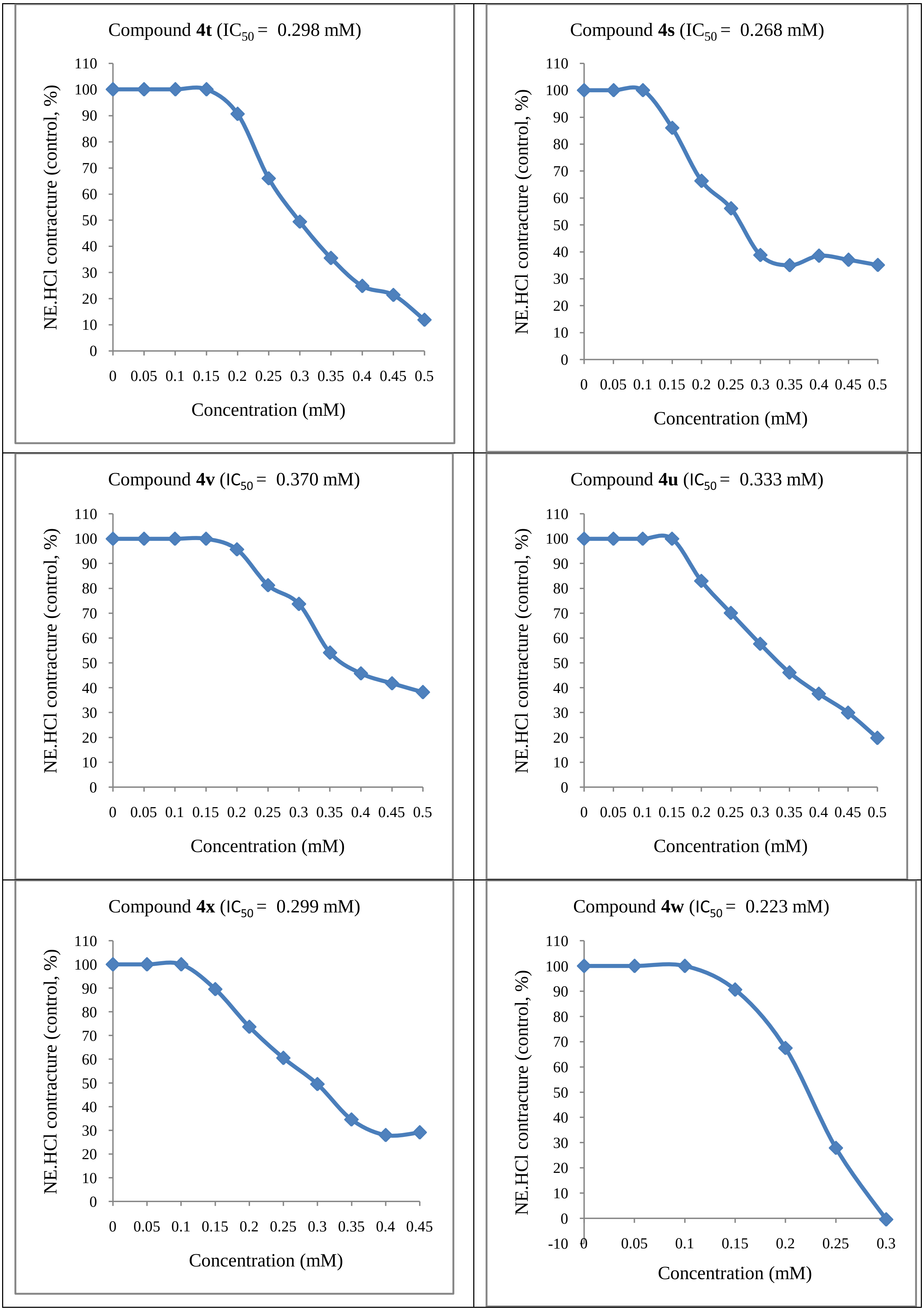
Compound 4t (IC50 = 0.298 mM)
NE.HCl contracture (control, %)
Concentration (mM)
Compound 4s (IC50 = 0.268 mM)
NE.HCl contracture (control, %)
Concentration (mM)
Compound 4v (IC50 = 0.370 mM)
NE.HCl contracture (control, %)
Concentration (mM)
Compound 4u (IC50 = 0.333 mM)
NE.HCl contracture (control, %)
Concentration (mM)
Compound 4x (IC50 = 0.299 mM)
NE.HCl contracture (control, %)
Concentration (mM)
Compound 4w (IC50 = 0.223 mM)
NE.HCl contracture (control, %)
Concentration (mM)

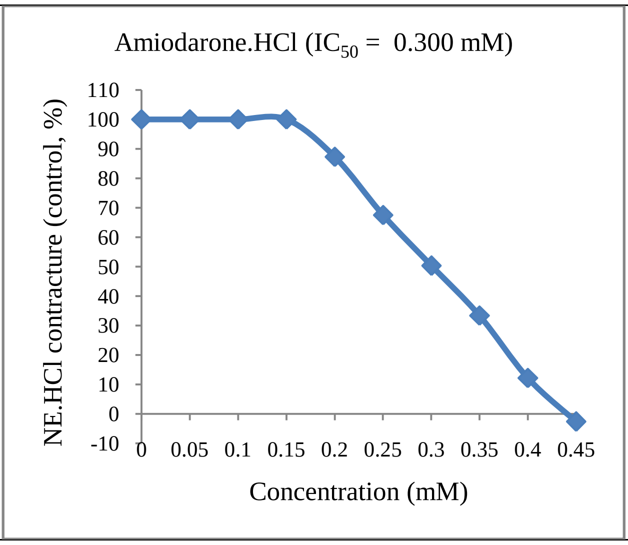


**Figure S1:** Effect of new chemical entities and the reference standard (Amiodarone hydrochloride) on contracture induced by norepinephrine hydrochloride (NE.HCl) in rat thoracic aortic rings.

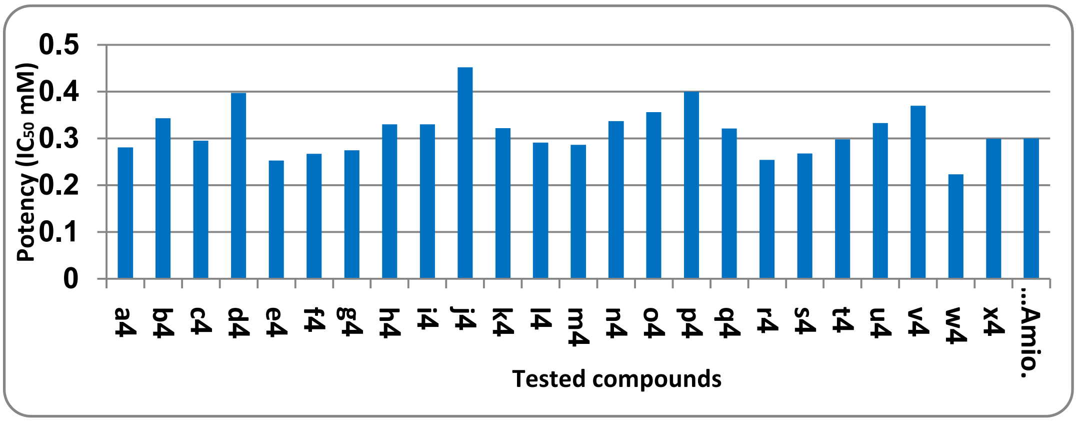


**Figure S2.** Potency ($IC_{50}$, mM) of the tested compounds on contracture induced by norepinephrine hydrochloride in rat thoracic aortic rings compared with (Amiodarone hydrochloride) used as a reference standard.

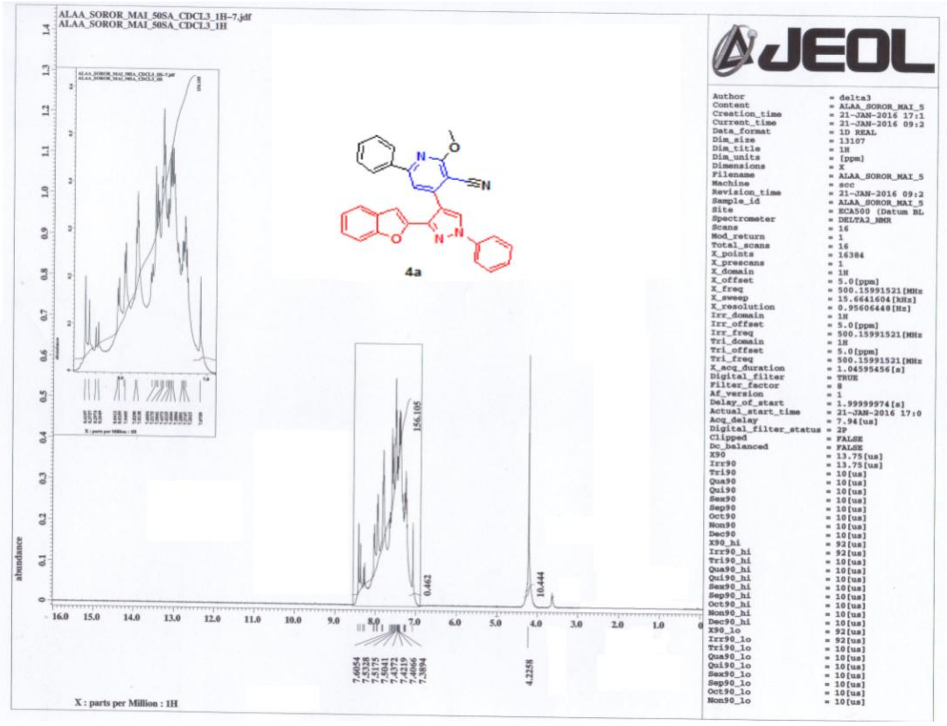

**Figure S3.** $^{1}$H NMR spectrum of **4a**.

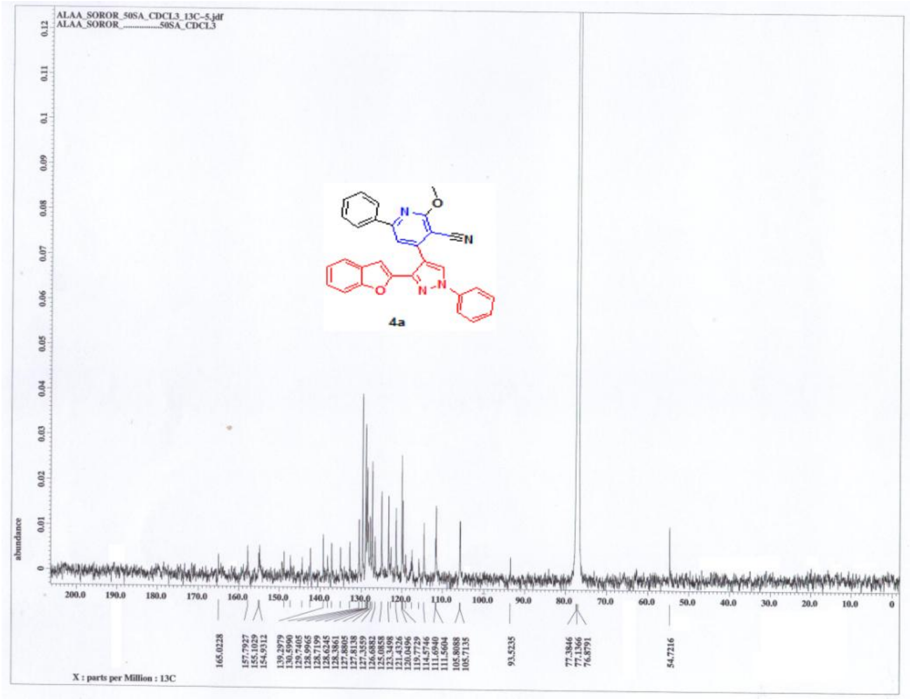

**Figure S4.** $^{13}$C NMR spectrum of **4a**.

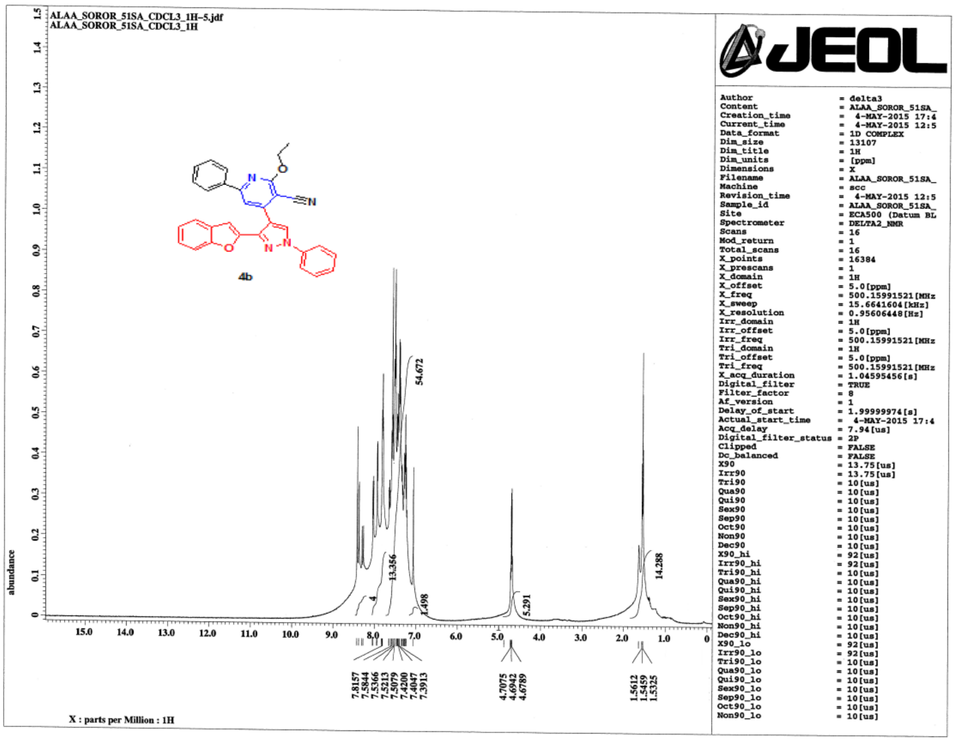

**Figure S5.** $^{1}$H NMR spectrum of **4b**.

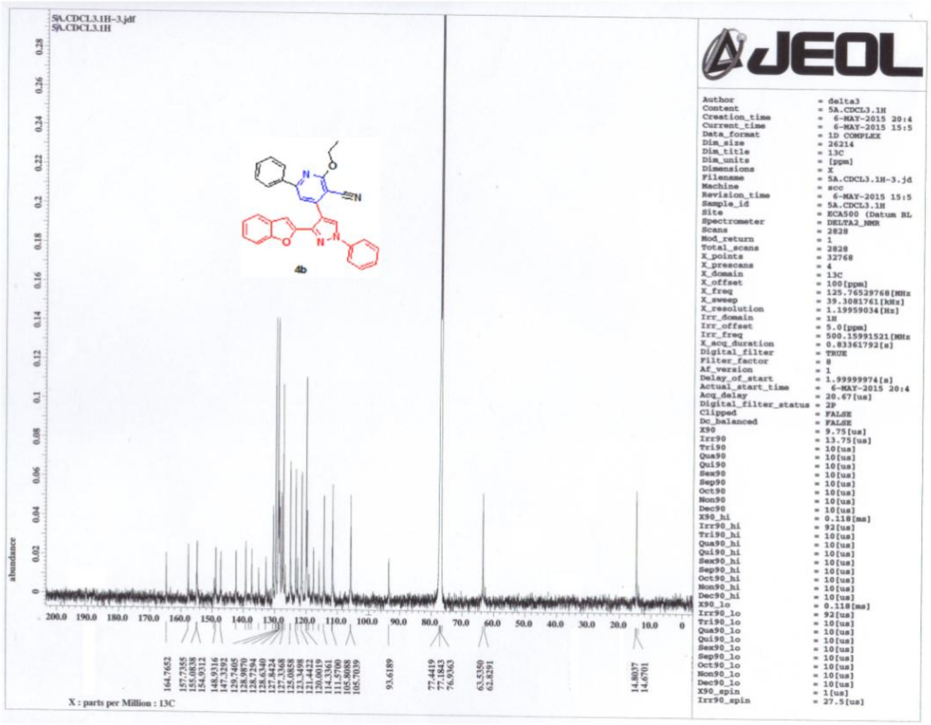

**Figure S6.** $^{13}$C NMR spectrum of **4b**.

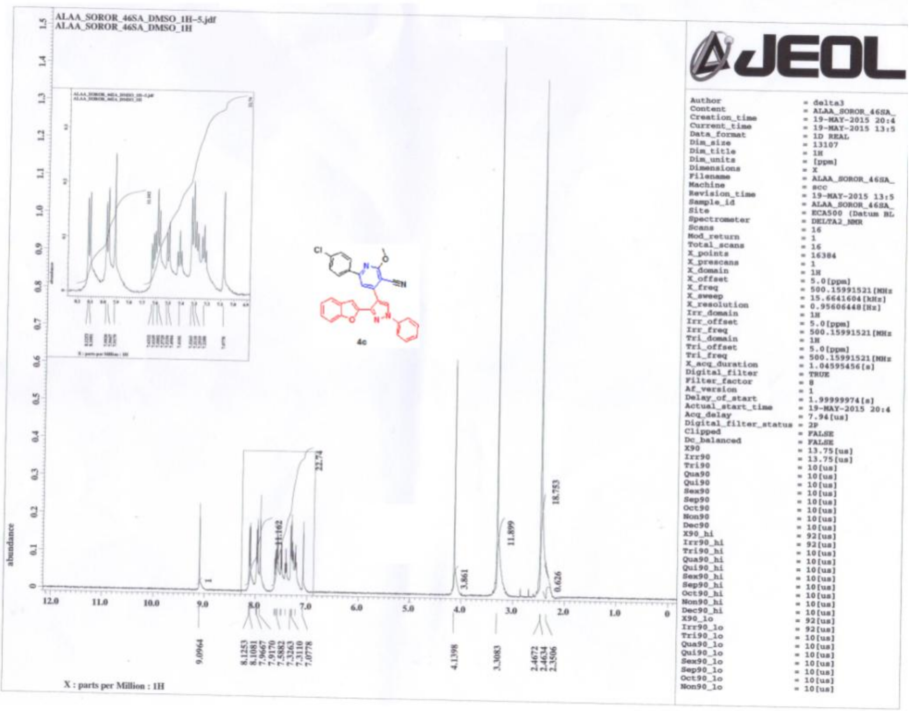

**Figure S7.** $^{1}$H NMR spectrum of **4c**.

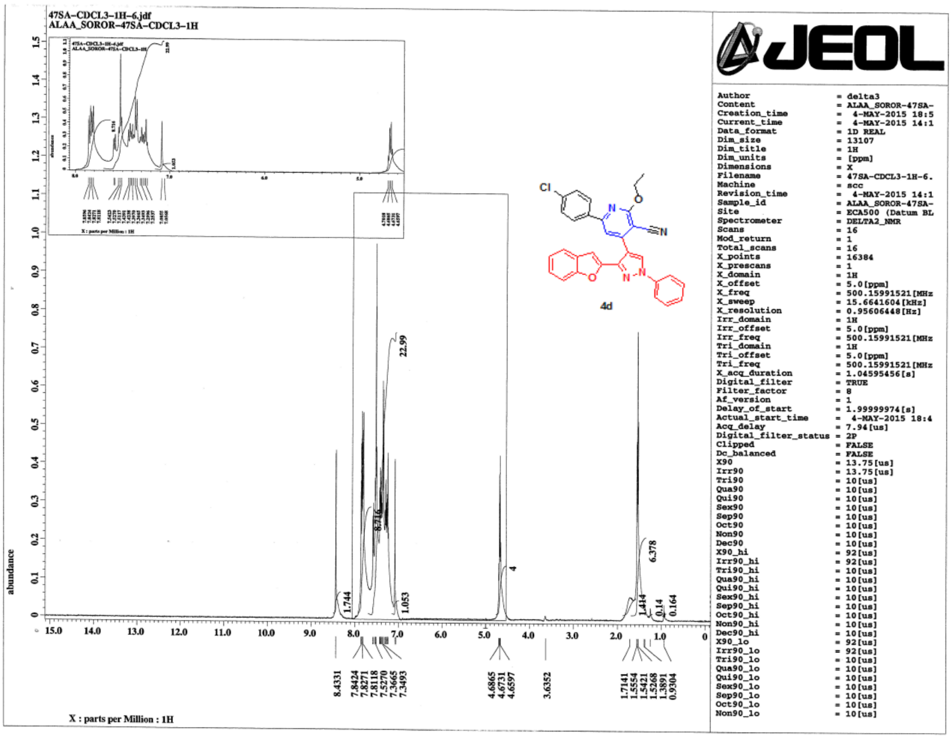

**Figure S8**. $^{1}$H NMR spectrum of **4d**.

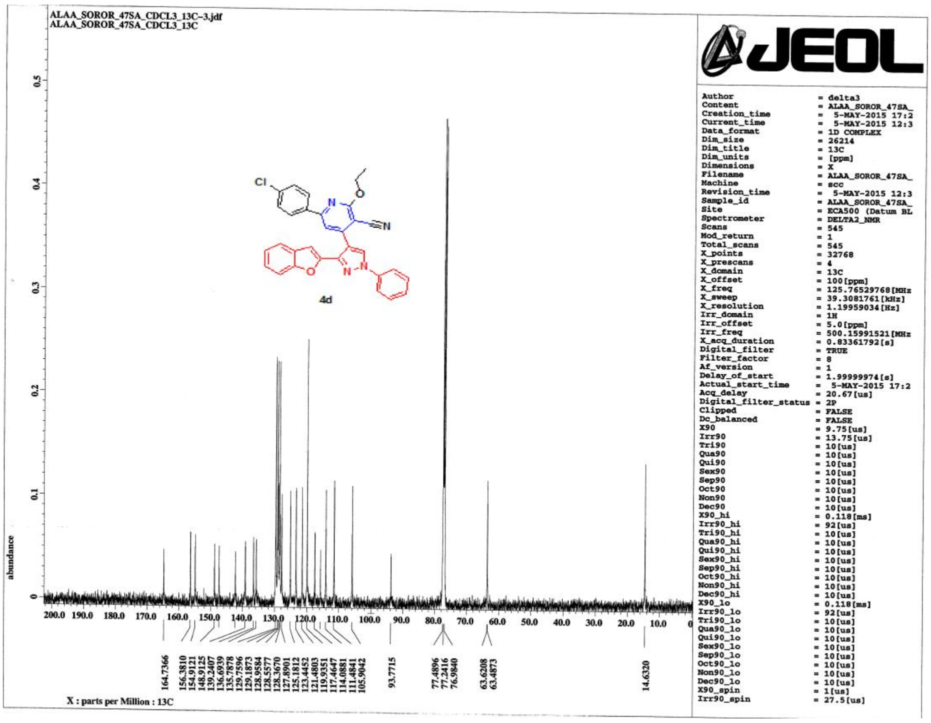


**Figure S9.** $^{13}$C NMR spectrum of **4d**.

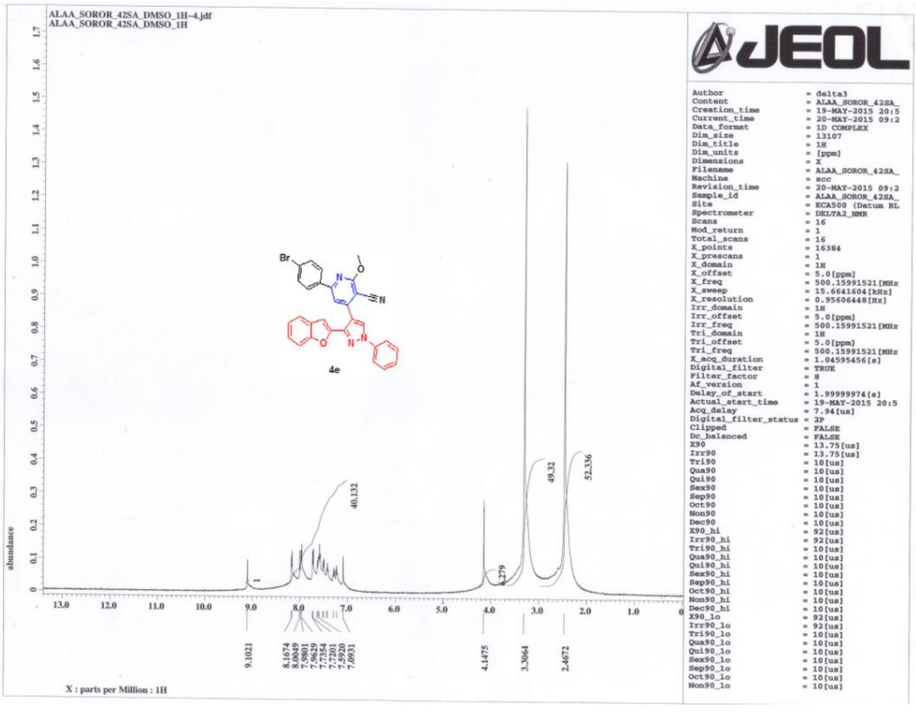


**Figure S10.** $^{1}$H NMR spectrum of **4e**.

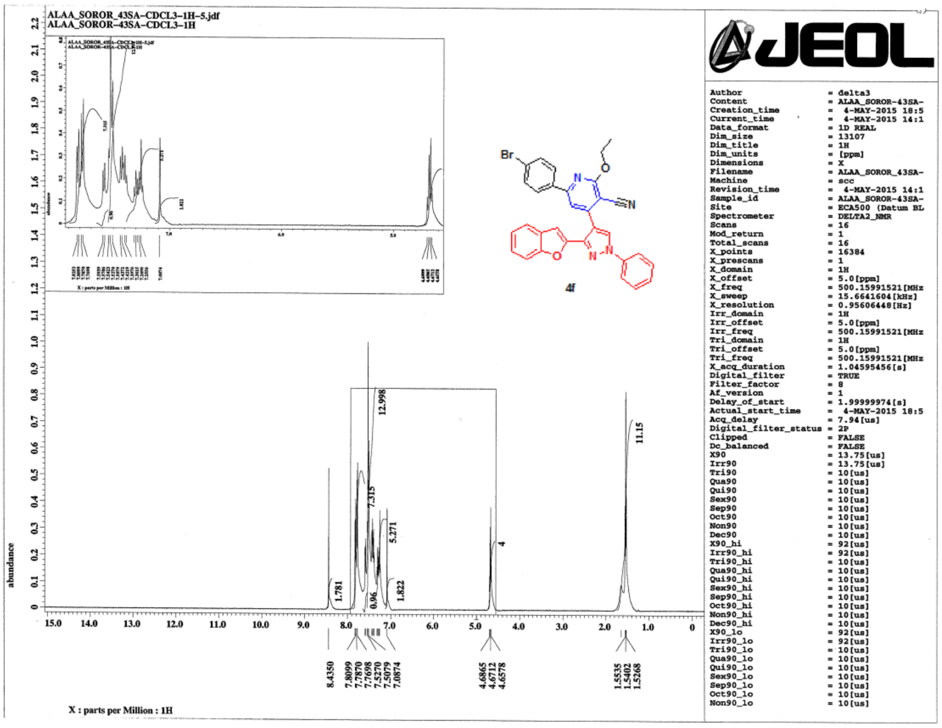

**Figure S11.** $^{1}$H NMR spectrum of **4f**.

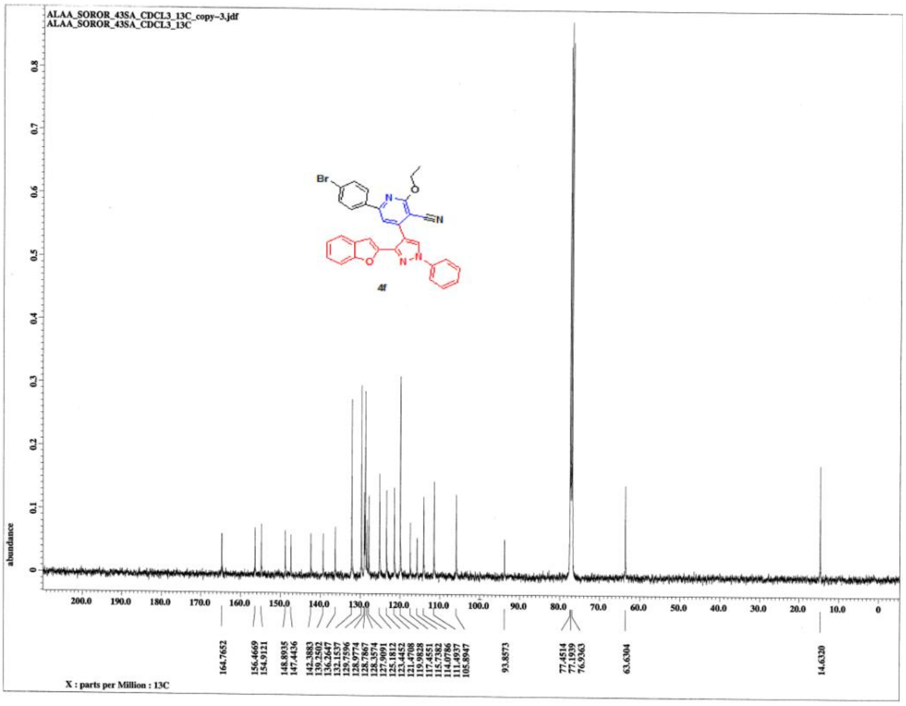

**Figure S12.** $^{13}$C NMR spectrum of **4f**.

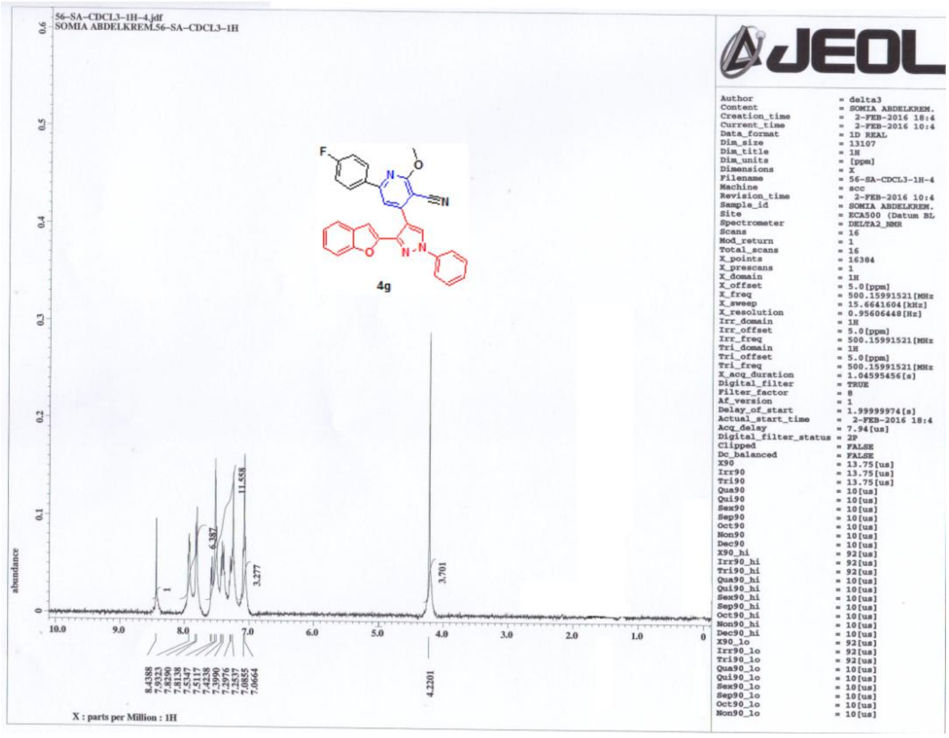

**Figure S13.** $^{1}$H NMR spectrum of **4g**.

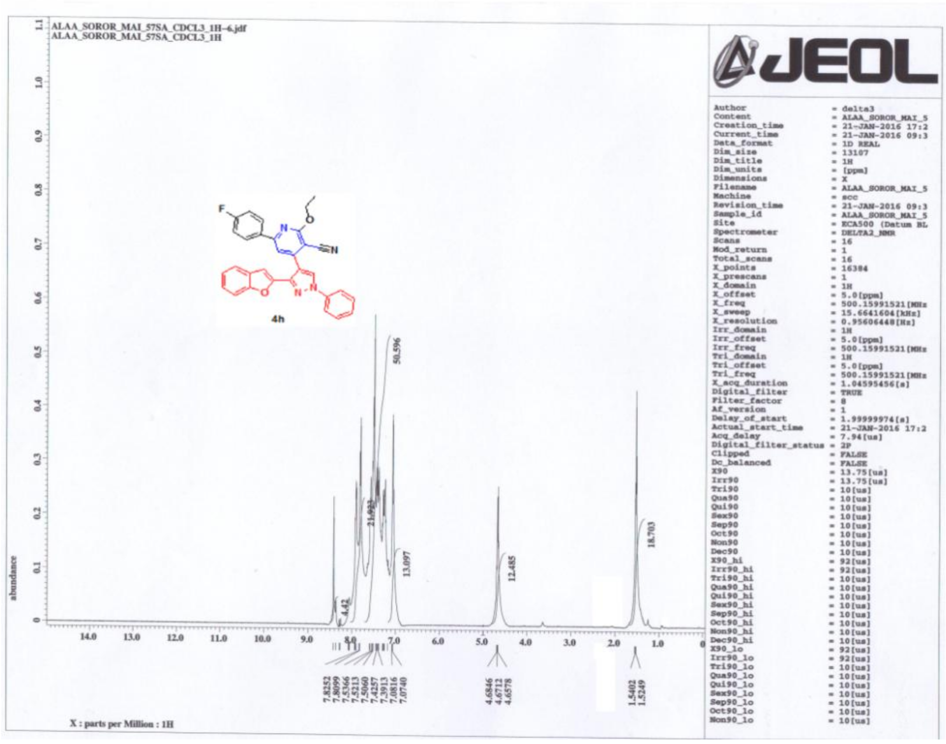

**Figure S14.** $^{1}$H NMR spectrum of **4h**.

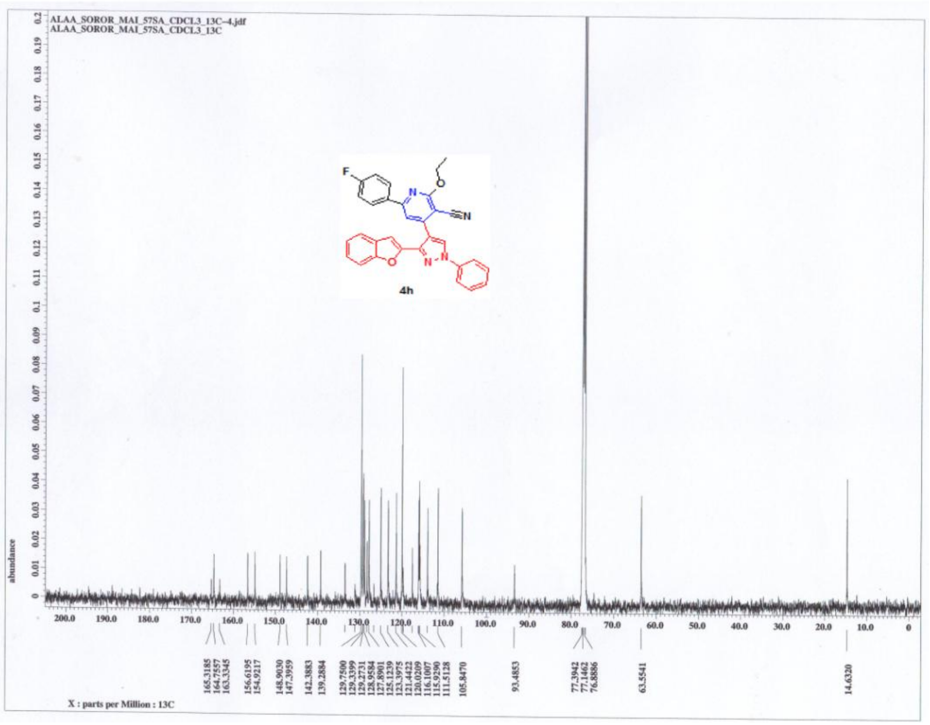


**Figure S15.** $^{13}$C NMR spectrum of **4h**.

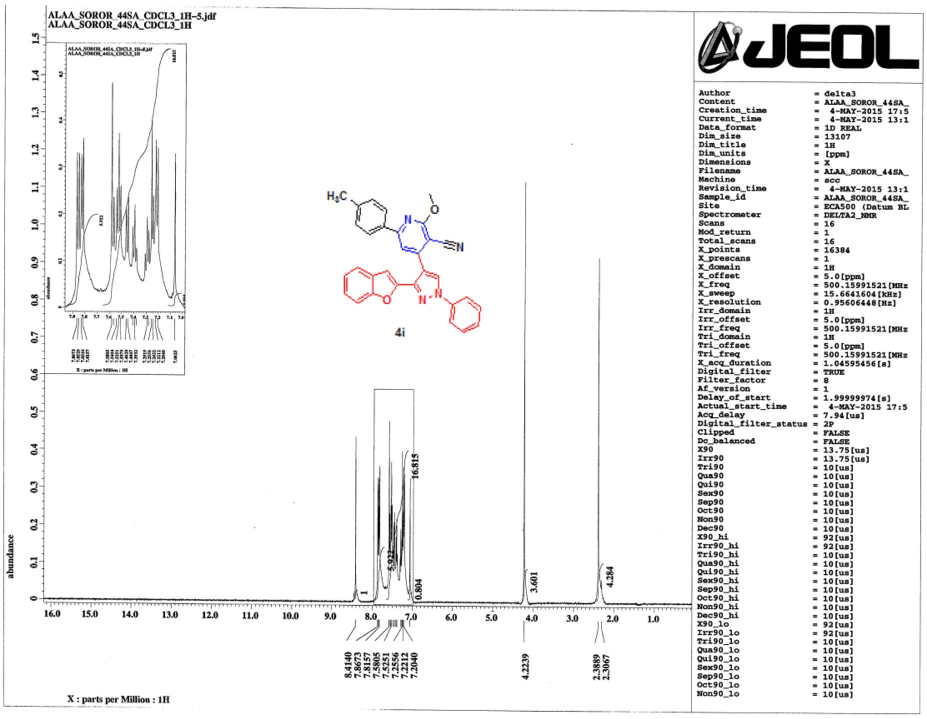


**Figure S16.** $^{1}$H NMR spectrum of **4i**.

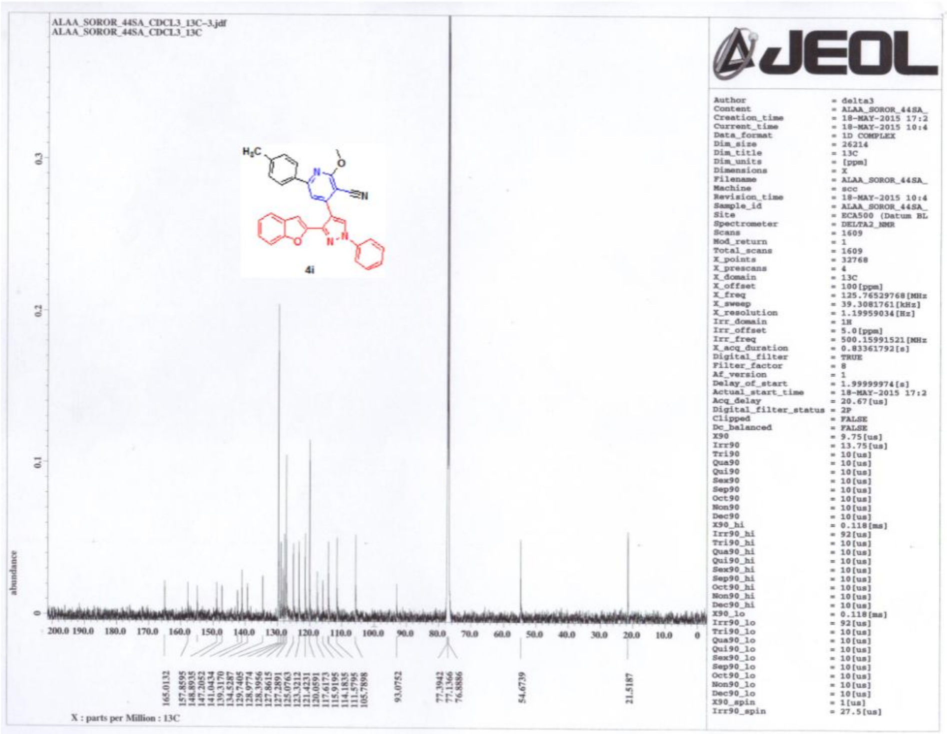

**Figure S17.** $^{13}C$ NMR spectrum of **4i**.

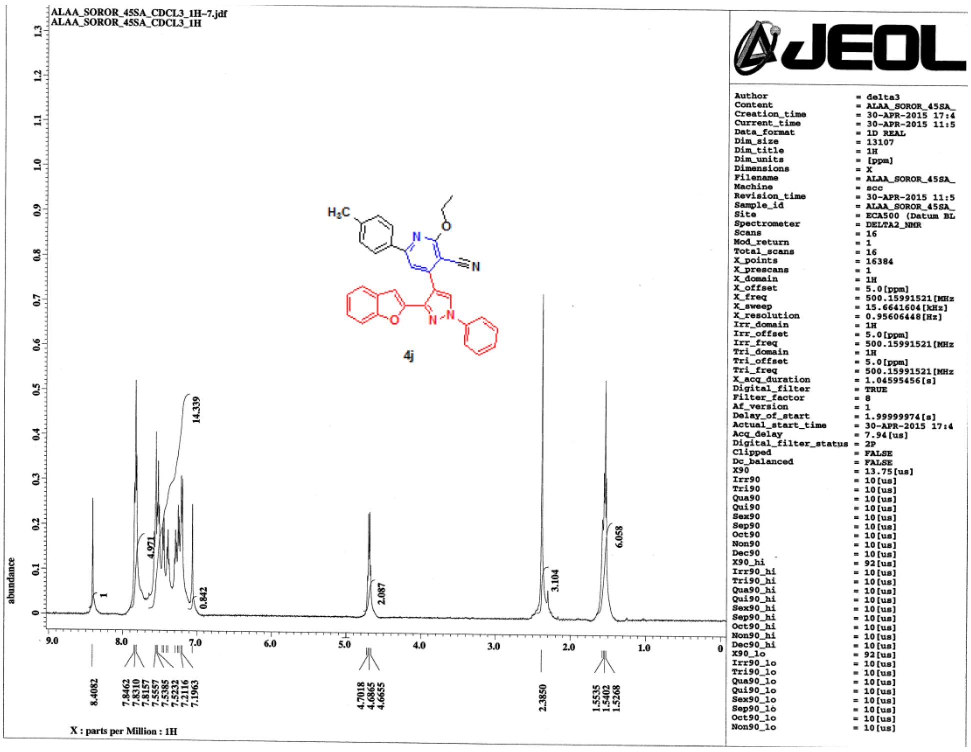

**Figure S18.** $^{1}H$ NMR spectrum of **4j**.

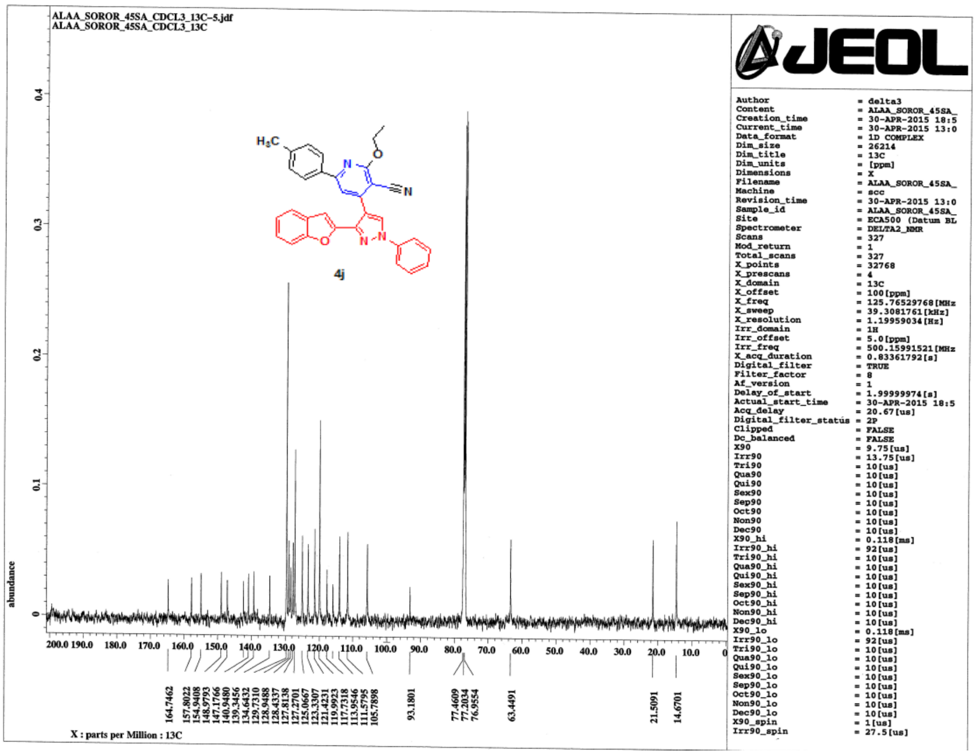

**Figure S19.** $^{13}$C NMR spectrum of **4j**.

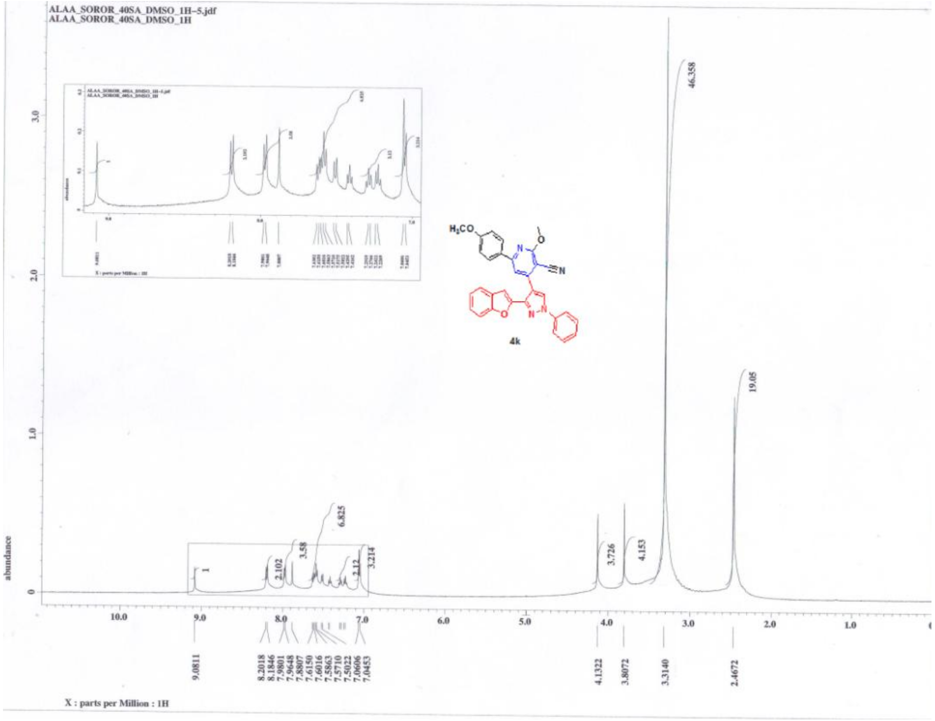

**Figure S20.** $^{1}$H NMR spectrum of **4k**.

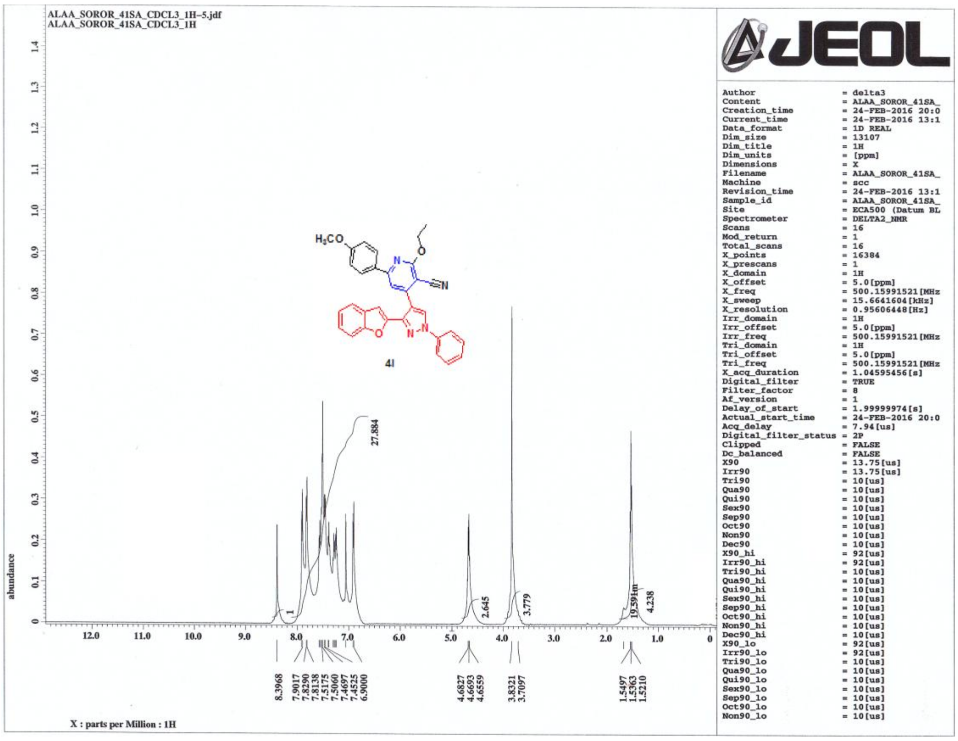



**Figure S21.** $^{1}$H NMR spectrum of **4l**.

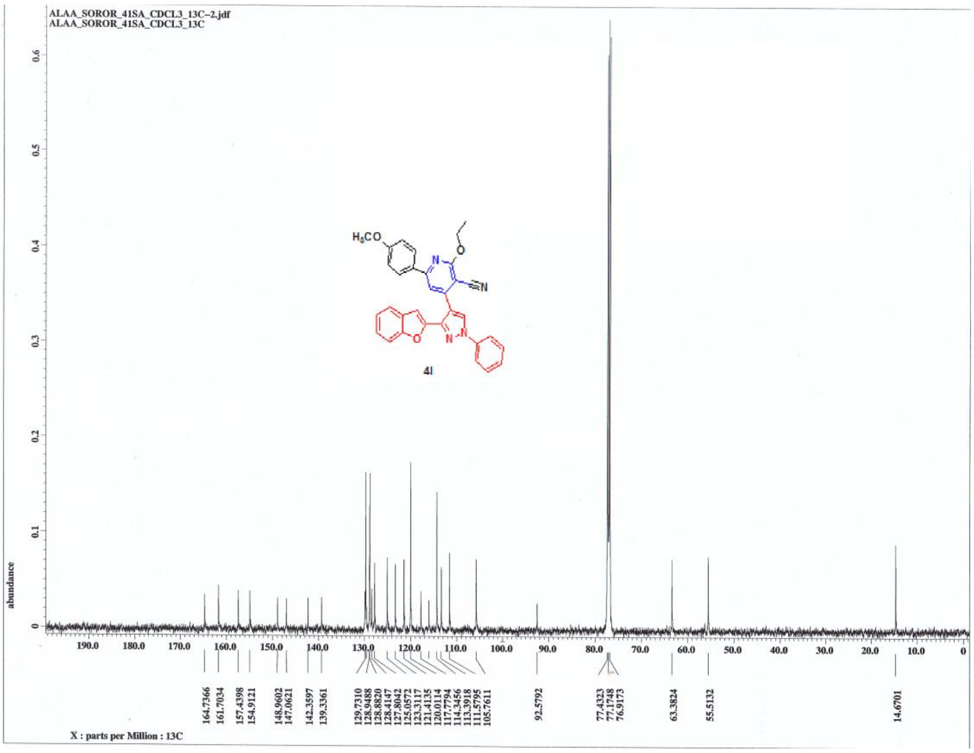



**Figure S22.** $^{13}$C NMR spectrum of **4l**.

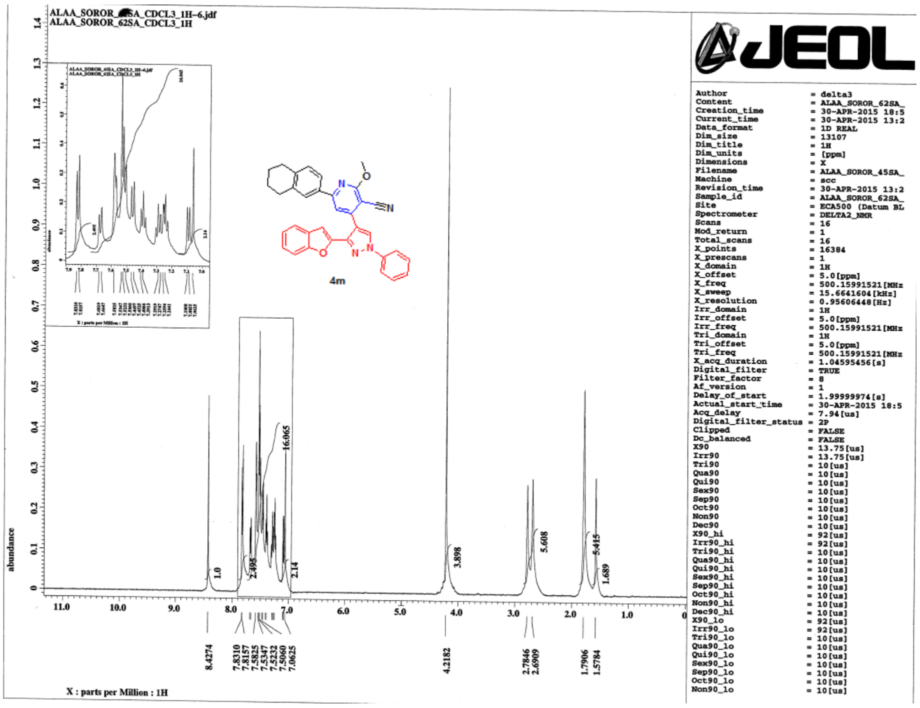

**Figure S23.** $^{1}$H NMR spectrum of **4m**.

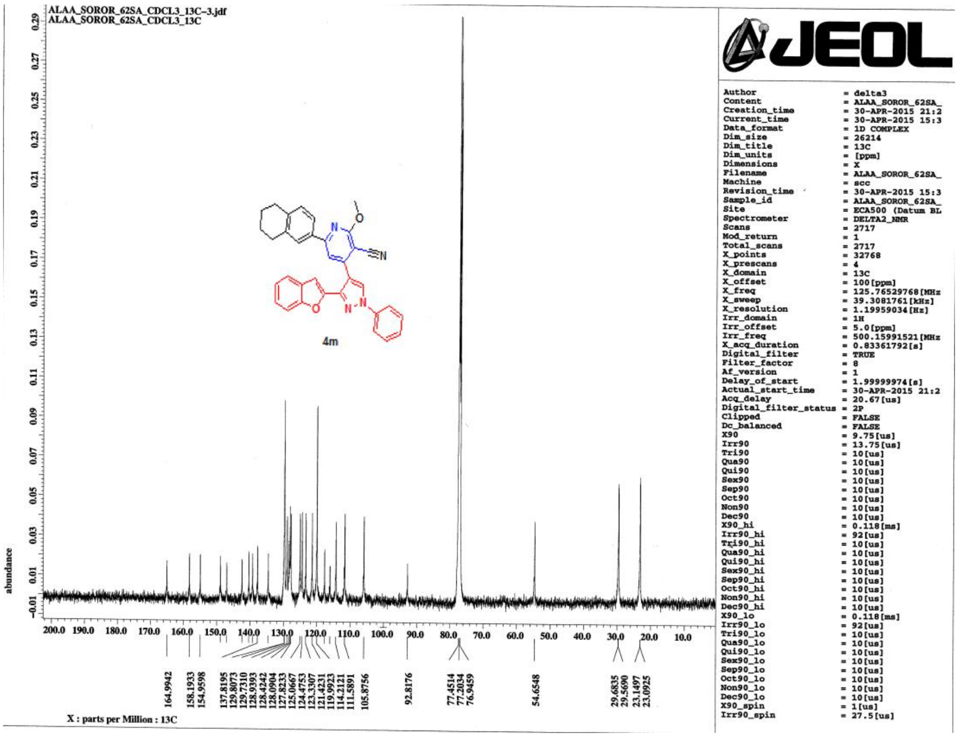

**Figure S24.** $^{13}$C NMR spectrum of **4m**.

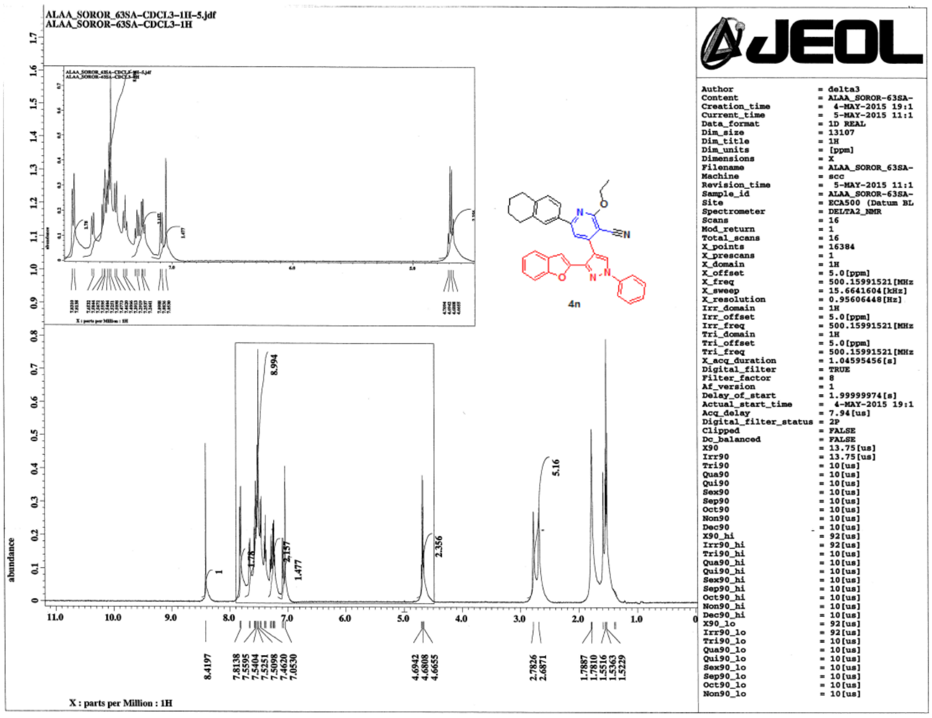

**Figure S25.** $^{1}H$ NMR spectrum of **4n**.

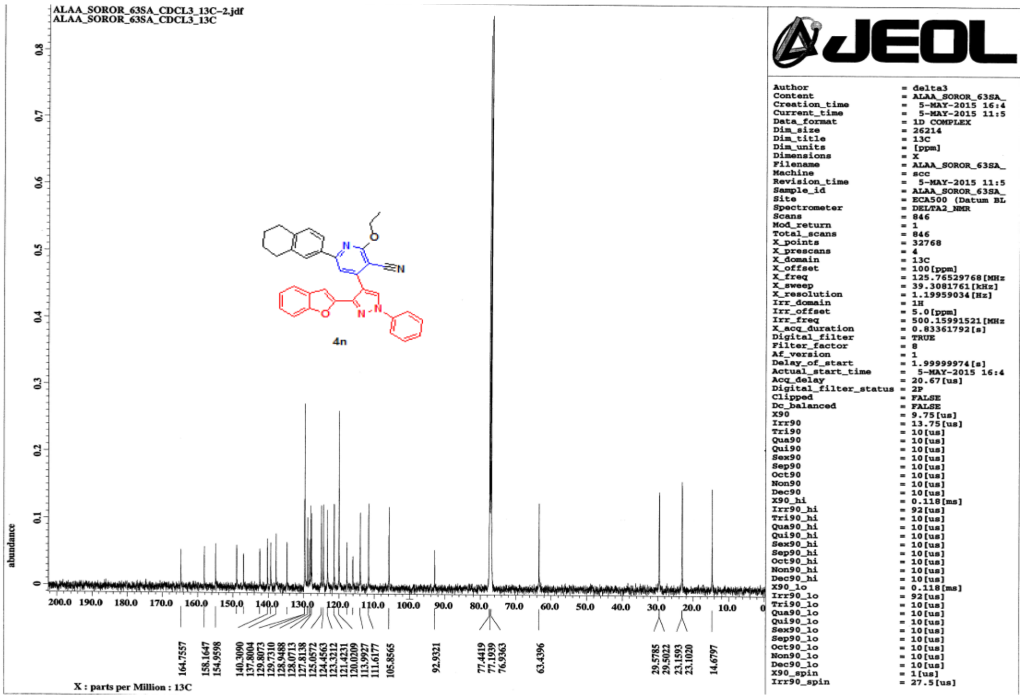

**Figure S26.** $^{13}C$ NMR spectrum of **4n**.

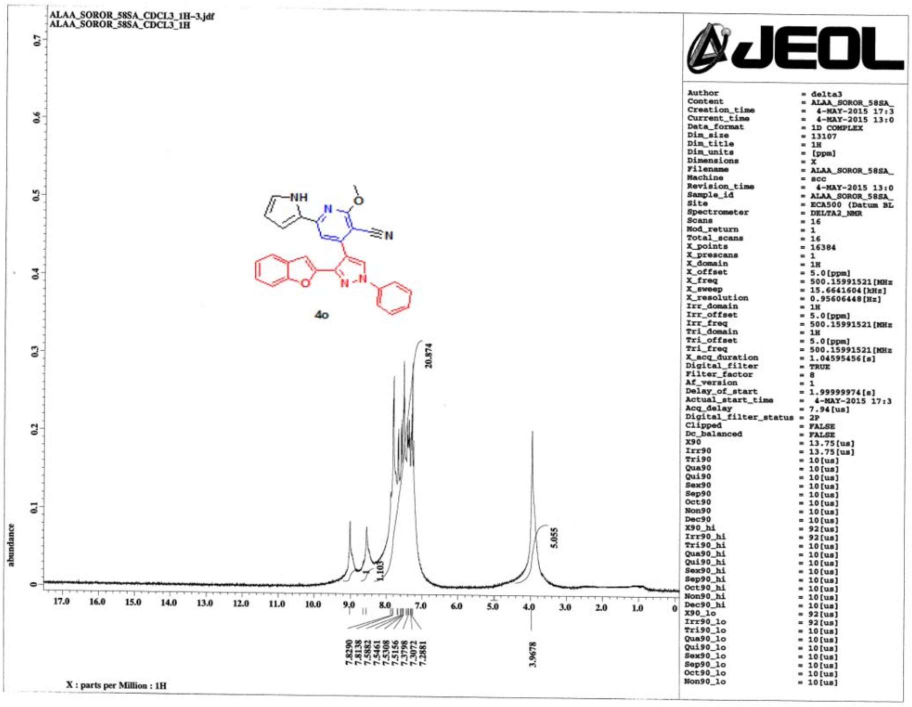


**Figure S27.** $^{1}$H NMR spectrum of **4m**.

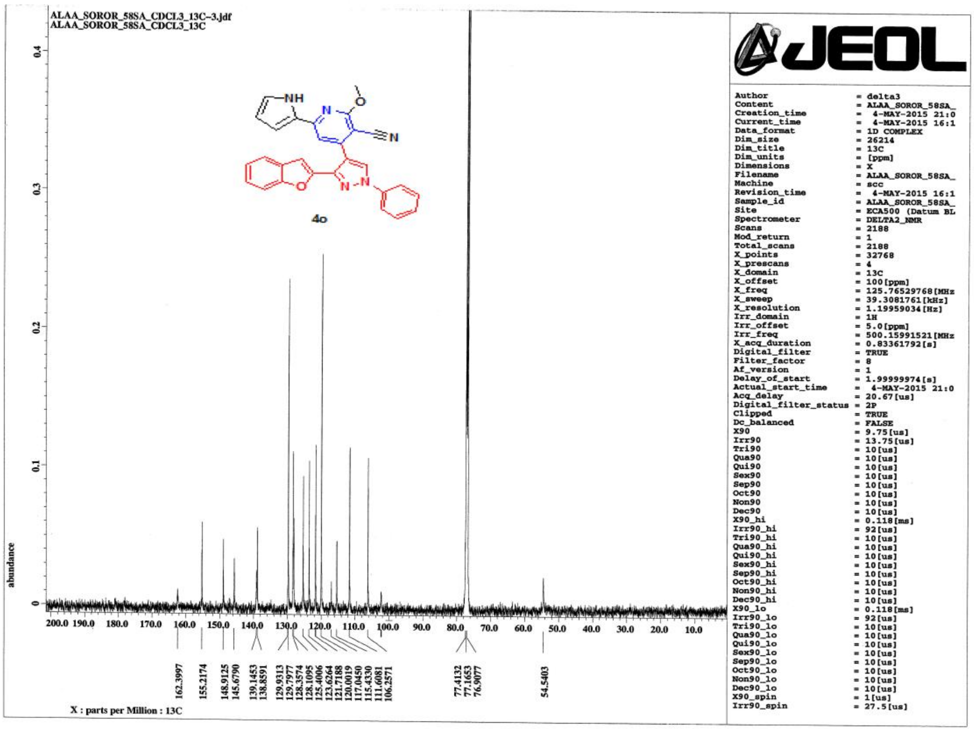


**Figure S28.** $^{13}$C NMR spectrum of **4o**.

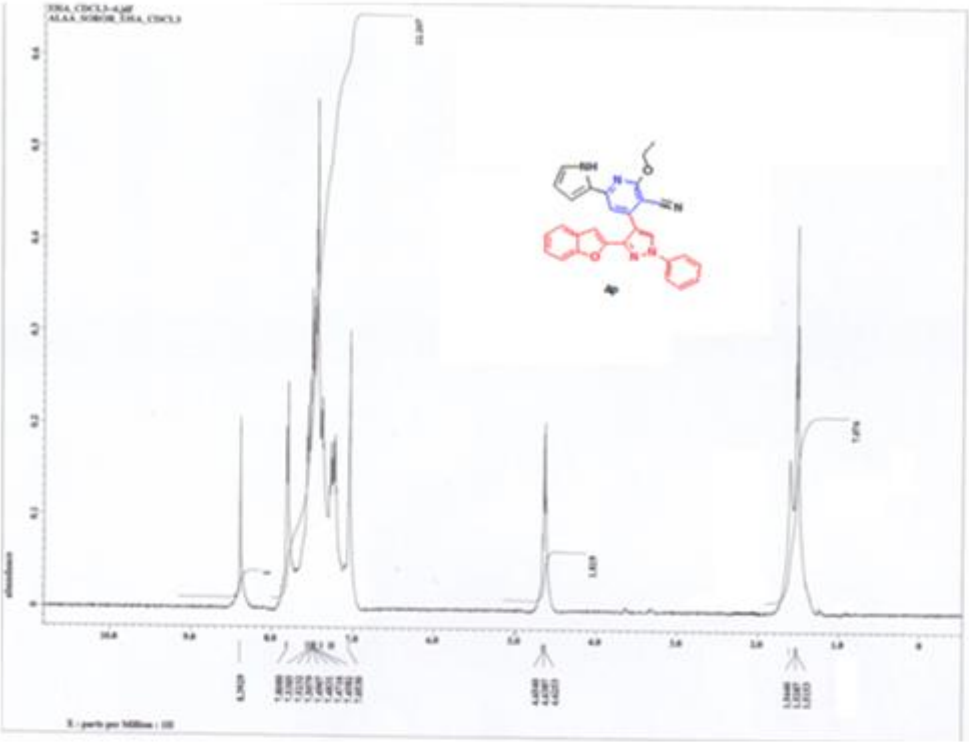

**Figure S29.** $^{1}H$ NMR spectrum of **4p**.

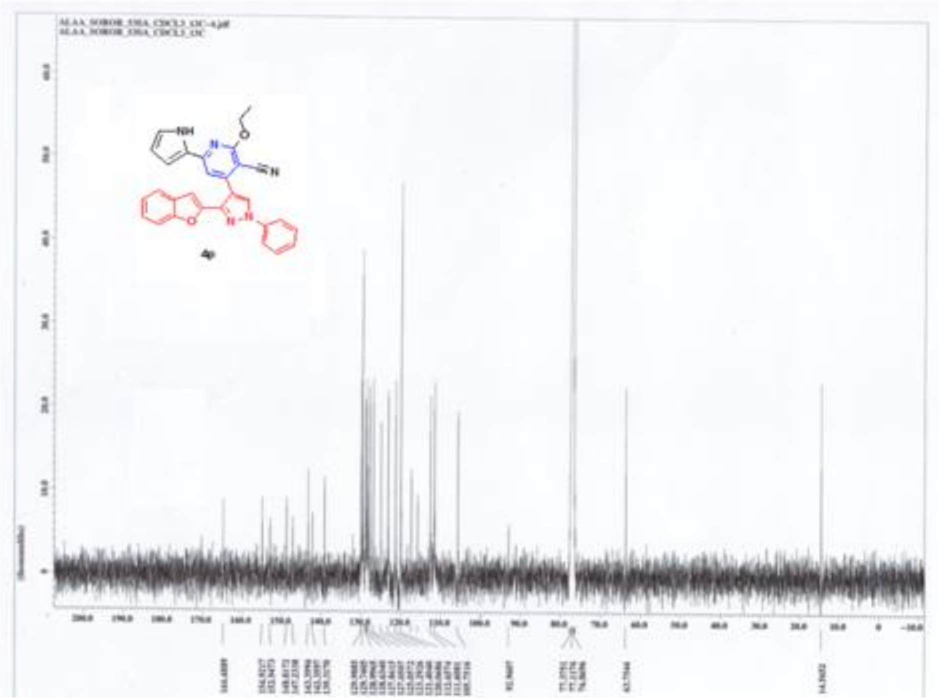

**Figure S30.** $^{13}C$ NMR spectrum of **4p**.

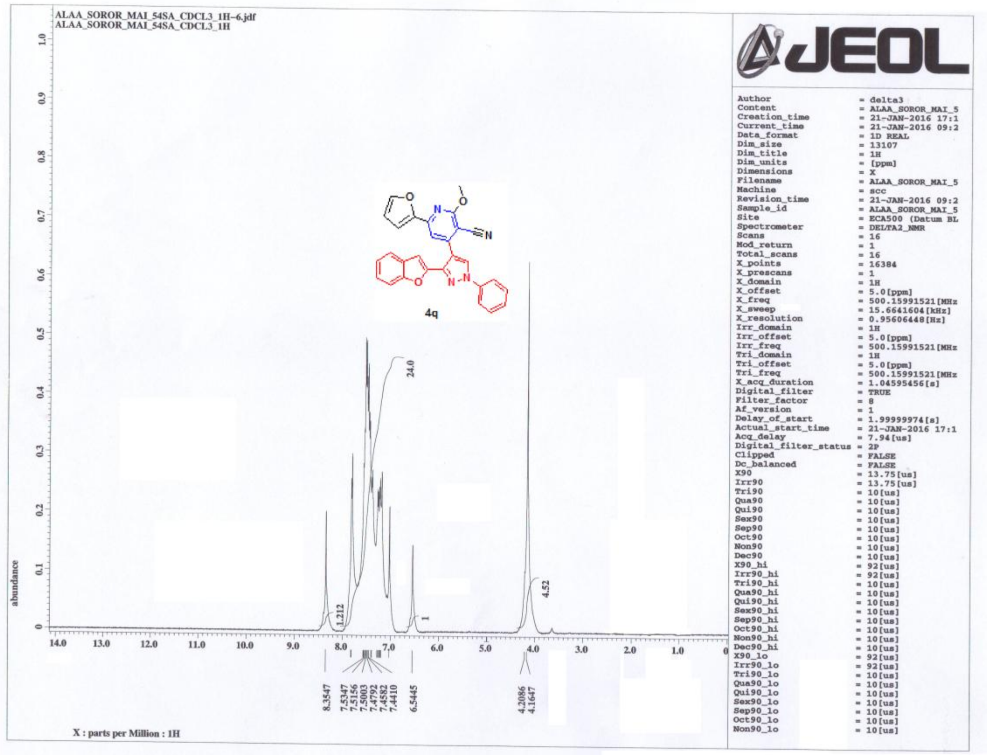


**Figure S31.** $^{1}$H NMR spectrum of **4q**.

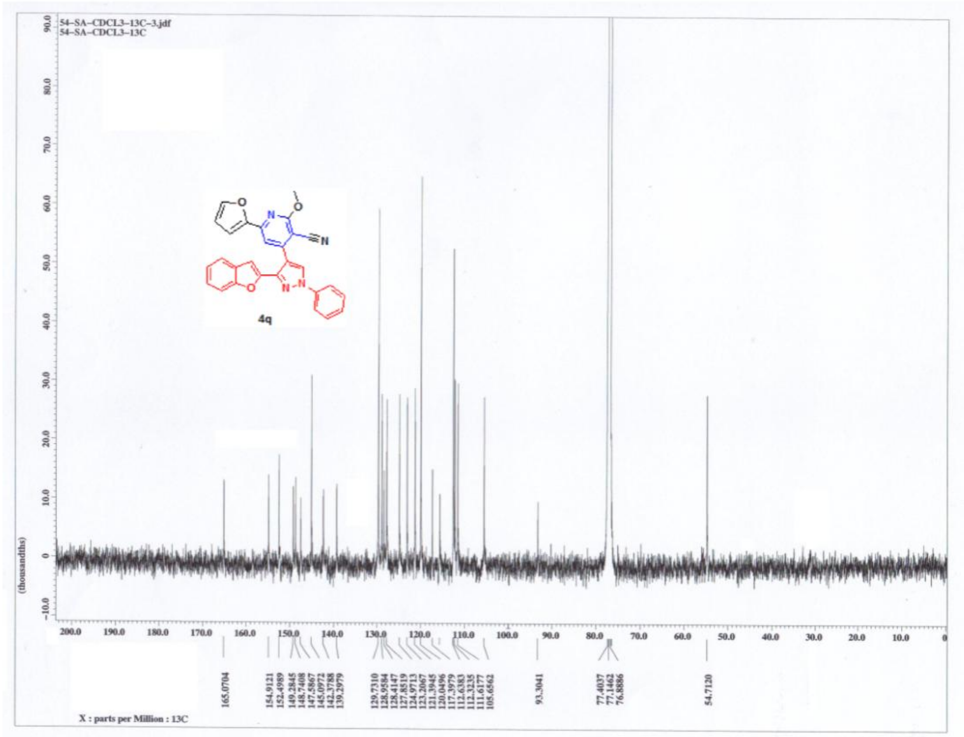


**Figure S32.** $^{13}$C NMR spectrum of **4q**.

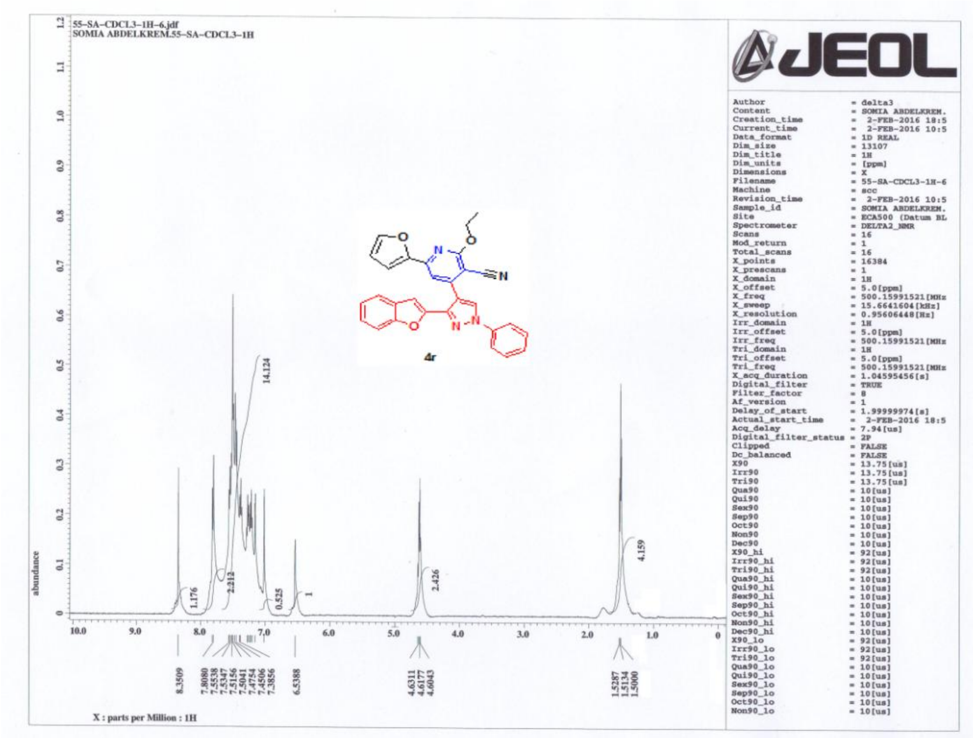


**Figure S33.** $^{1}$H NMR spectrum of **4r**.

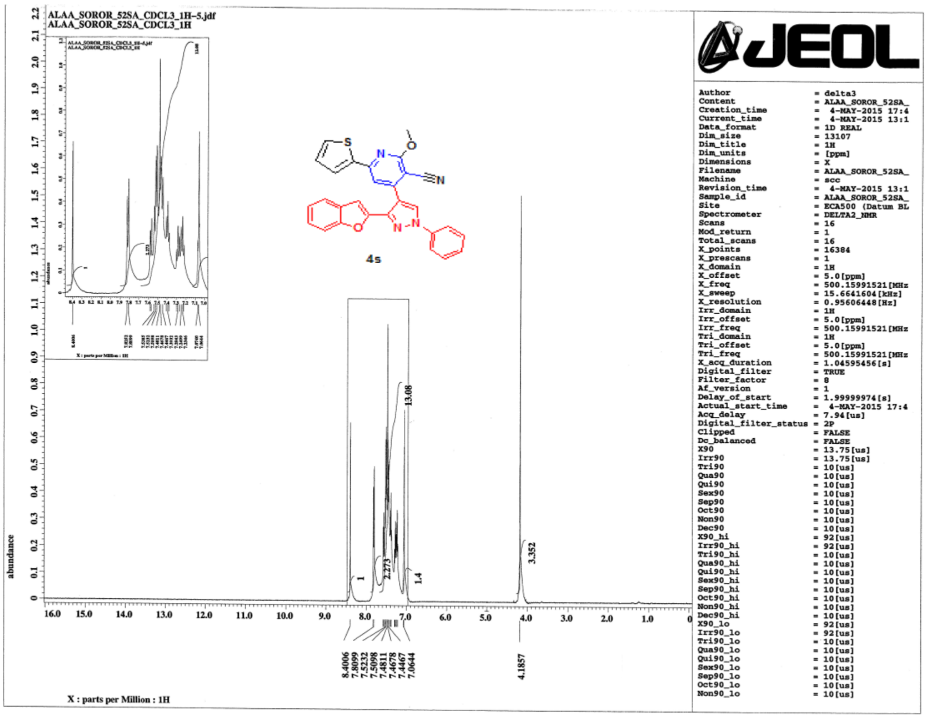


**Figure S34.** $^{1}$H NMR spectrum of **4s**.

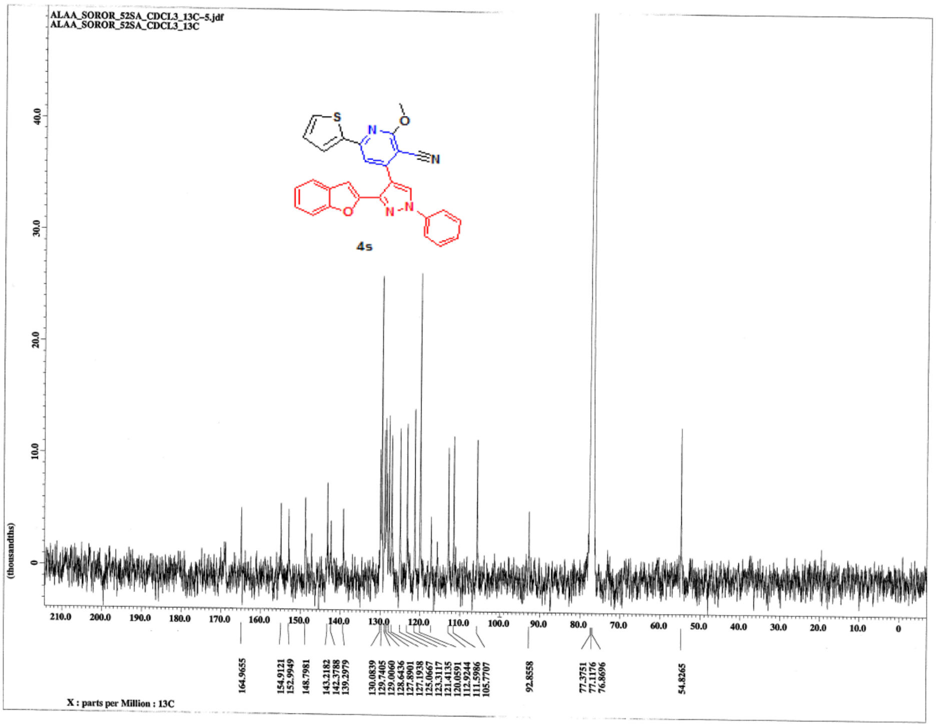


**Figure S35.** $^{13}$C NMR spectrum of **4s**.

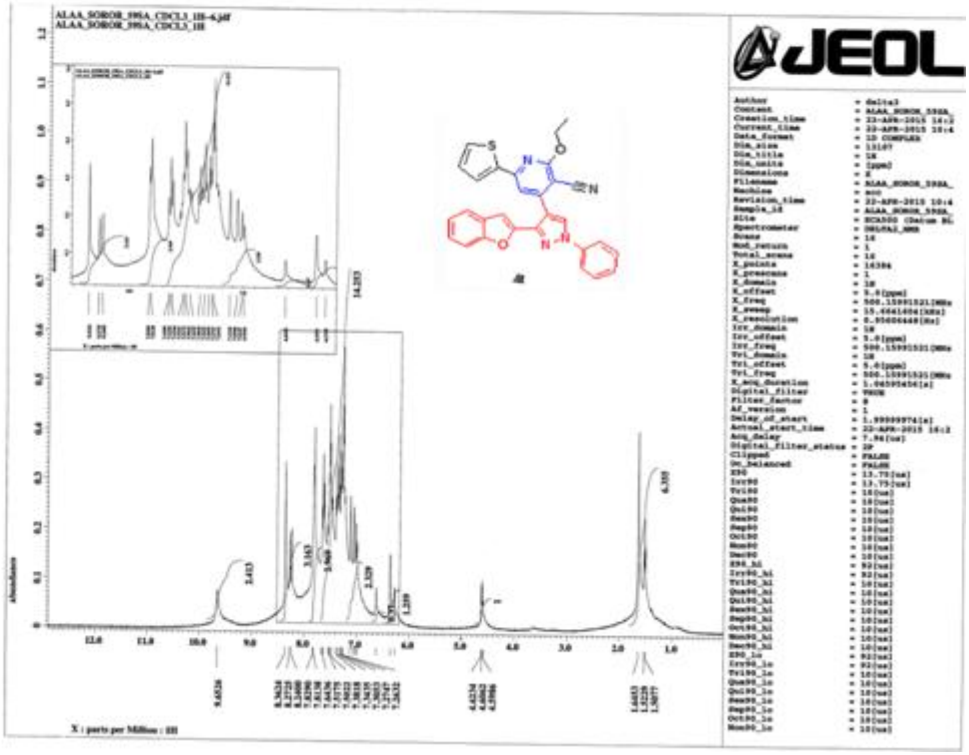


**Figure S36.** $^{1}$H NMR spectrum of **4t**.

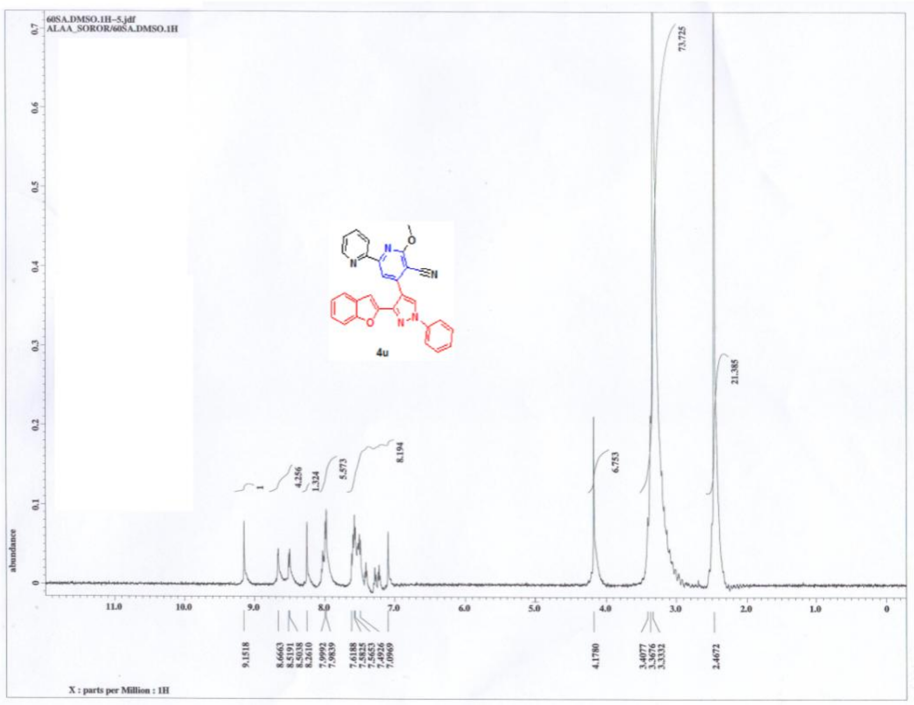

**Figure S37.** $^1$H NMR spectrum of **4u**.

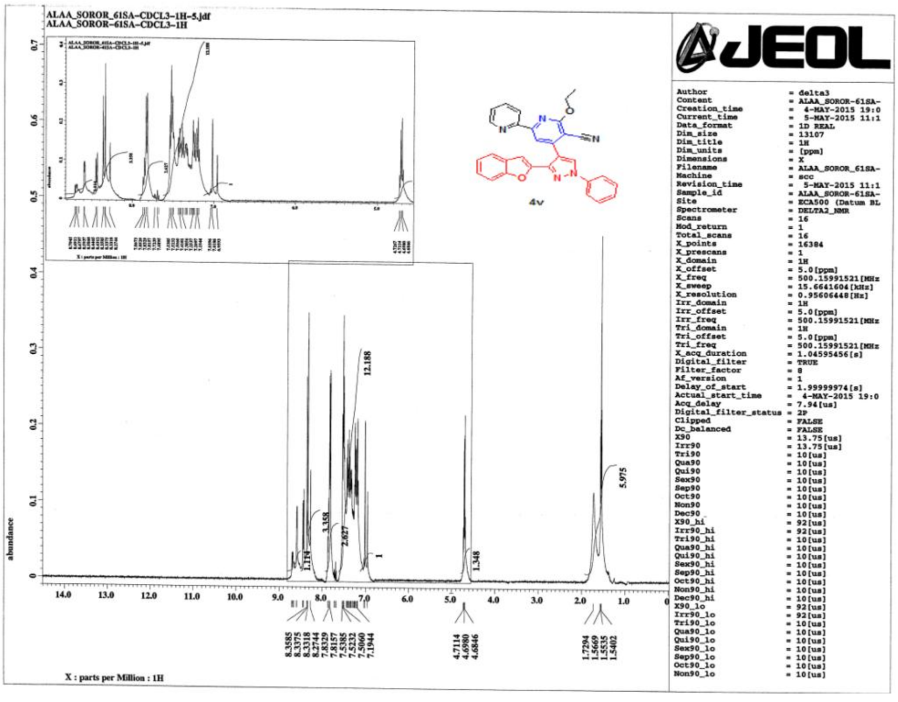

**Figure S38.** $^1$H NMR spectrum of **4v**.

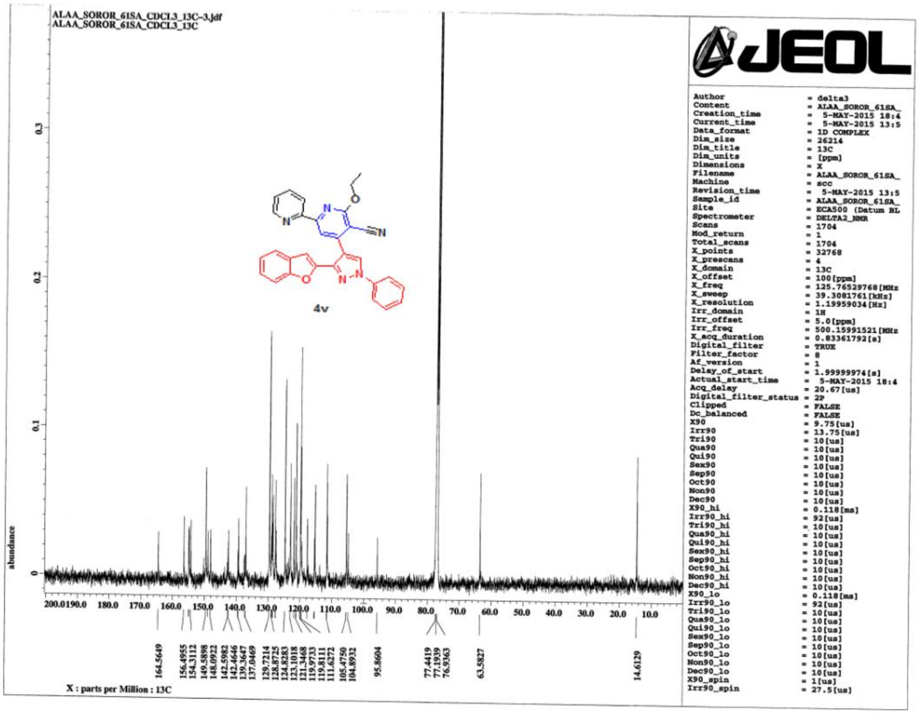


**Figure S39.** $^{13}$C NMR spectrum of **4v**.

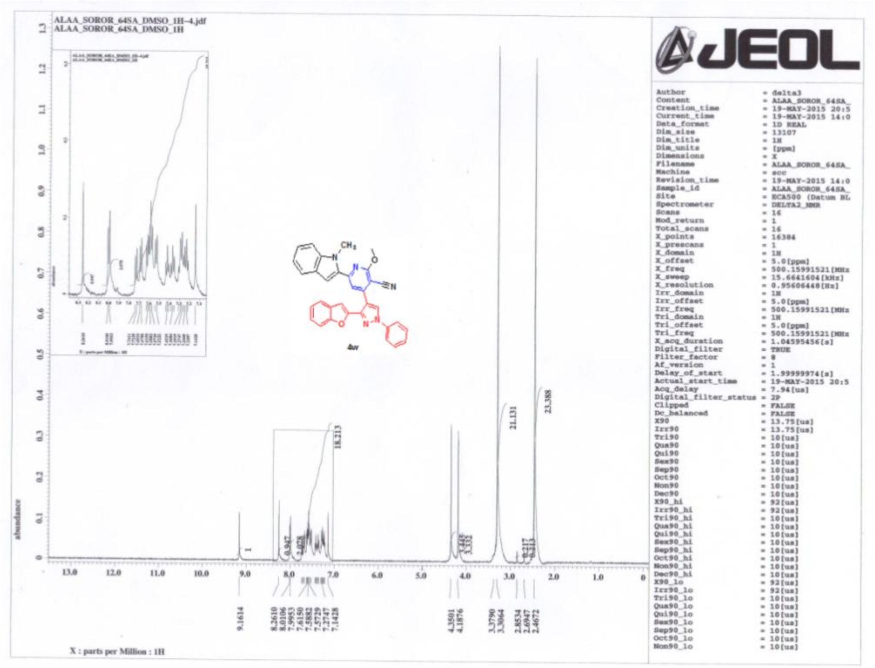


**Figure S40.** $^{1}$H NMR spectrum of **4w**.

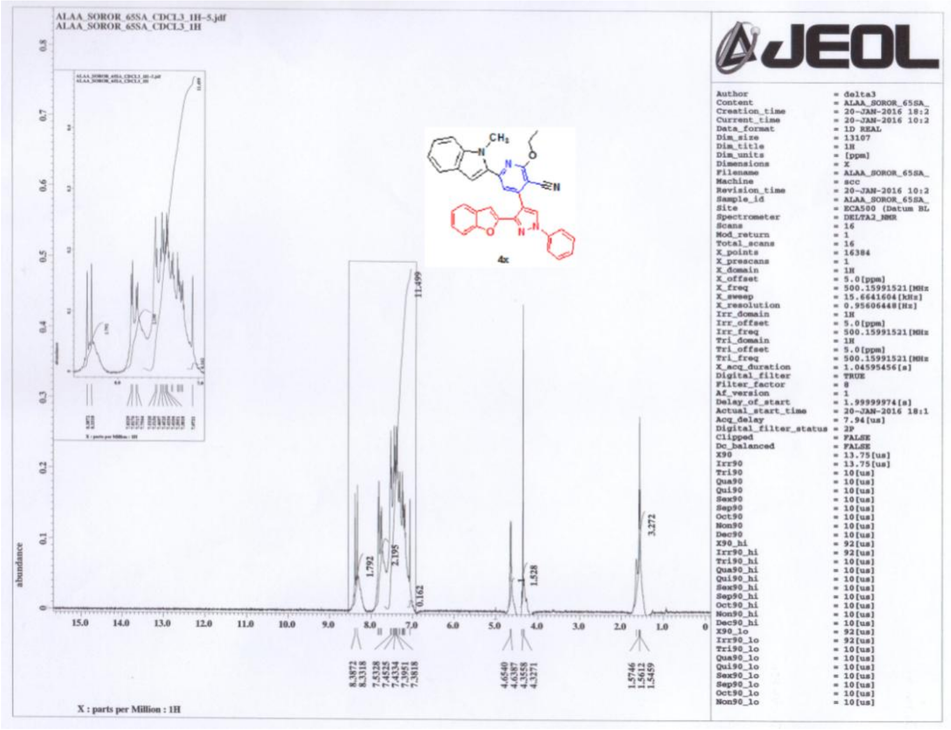

**Figure S41.** $^{1}$H NMR spectrum of **4x**.

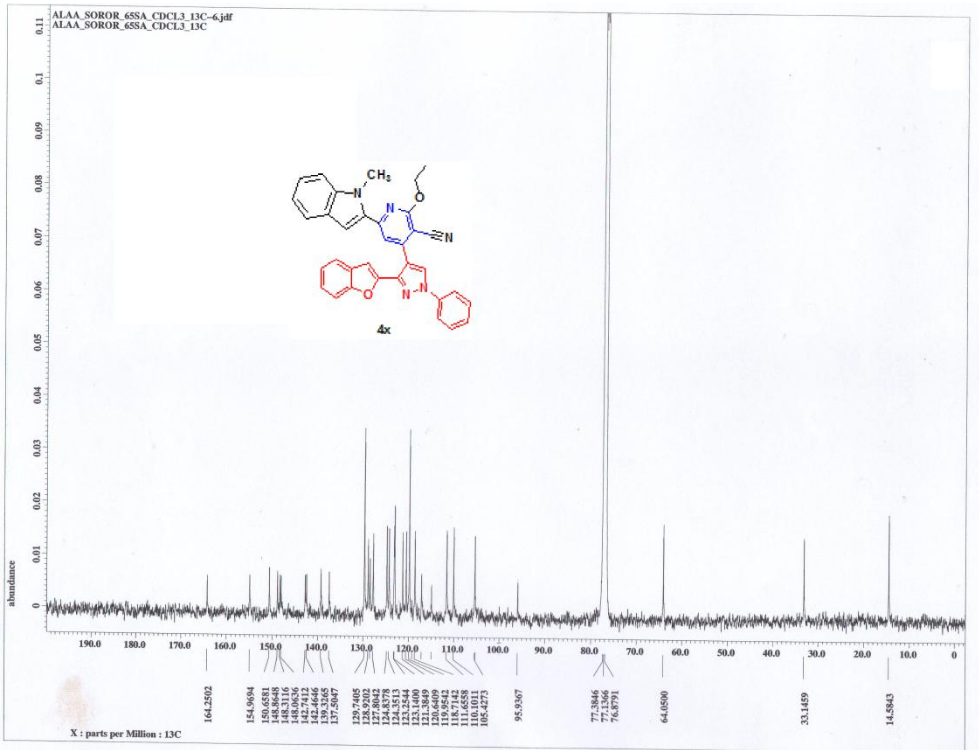

**Figure S42.** $^{13}$C NMR spectrum of **4x**.
